# Continuous flow photolysis of aryl azides: Preparation of 3*H*-azepinones

**DOI:** 10.3762/bjoc.7.129

**Published:** 2011-08-17

**Authors:** Farhan R Bou-Hamdan, François Lévesque, Alexander G O'Brien, Peter H Seeberger

**Affiliations:** 1 Max Planck Institute of Colloids and Interfaces, Department of Biomolecular Systems, Am Mühlenberg 1, 14476 Potsdam, Germany and Freie Universität Berlin, Institute for Chemistry and Biochemistry, Arnimallee 22, 14195 Berlin, Germany

**Keywords:** azepinones, azides, continuous flow, nitrenes, photochemistry

## Abstract

Photolysis of aryl azides to give nitrenes, and their subsequent rearrangement in the presence of water to give 3*H*-azepinones, is performed in continuous flow in a photoreactor constructed of fluorinated ethylene polymer (FEP) tubing. Fine tuning of the reaction conditions using the flow reactor allowed minimization of secondary photochemical reactions.

## Findings

Although photochemical rearrangements are an important class of reactions for heterocycle synthesis [1–2], their use is often hindered by technical difficulties, both in research laboratories and in industry, particularly when large quantities of material are required [3–4]. In response, there has been a recent growth in the application of continuous flow techniques [5–7] for the deployment of photochemical reactions [8–13]. As, from the Beer–Lambert law, the intensity of light decreases exponentially with increasing distance from the light source, minimization of the path length in a continuous flow photoreactor ensures efficient and uniform irradiation of the sample [14], and scaling up is simple to achieve by running the reactor over an extended period. Additionally, the precise control of the reaction conditions and the continuous removal of products inherent in flow systems can offer improved yields and selectivities [8,15].

**Scheme 1:**
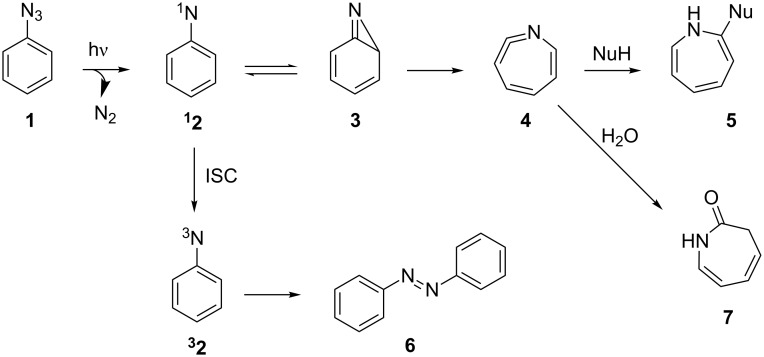
Products of aryl azide photolysis.

Nitrenes generated by aryl azide photolysis are important tools, both for preparative heterocycle synthesis [16–17] and for photoaffinity labeling of proteins [18–20]. The photolysis of aryl azide **1** [21], a well-studied and widely used reaction [22–30], generates the singlet aryl nitrene intermediate **^1^2** (Scheme 1). Ring expansion of **^1^2**, via 2*H*-azirine **3**, affords didehydroazepine **4**, which can be trapped by variety of nucleophiles to provide the corresponding azepine **5**. Alternatively, intersystem crossing (ISC) of **^1^2** gives rise to **^3^2**, which can dimerize to form diazo compound **6**. Performing the reaction in the presence of water typically affords 3*H*-azepinone **7** [31–34], a moiety present in natural products [35–36] and in azepinone-derived pharmaceuticals [37–38]. Despite the importance of these 7-membered nitrogen-containing heterocycles, there remain few methods for their preparation [39–44]. Overall, aryl azides, which are simple to prepare from the corresponding aniline derivative, are convenient precursors for the synthesis of azepine derivatives. However, the utility of the process is offset by the long reaction times required and by the low yields arising from poor selectivity and decomposition of the reaction mixture. We report herein the development of a continuous flow variant of the process and show how the reaction can be further optimized using a flow system [45–46].

For this study a 14 mL photoreactor was used, constructed from fluorinated ethylene polymer (FEP) tubing, wrapped around a 450 W medium pressure Hg lamp, surrounded by a cooling jacket and Pyrex filter (see Supporting Information File 1). Continuous flow photochemical reactors based upon FEP tubing are simple to construct and are seeing increasing use in photochemical synthesis [47–49]. A recirculating cryostat maintained an apparatus temperature of 25 °C for all experiments, and a back pressure regulator (6.9 bar) was used to suppress the formation of a separate gaseous phase from the evolved nitrogen. THF and water were pumped simultaneously into the reactor at the specified rates through a commercially available Vapourtec R2 pump, [50] and mixed by a PTFE T-mixer.

**Scheme 2:**
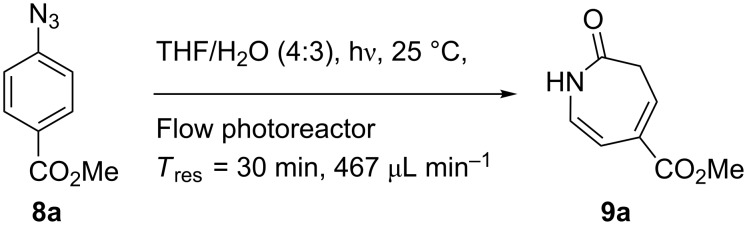
Optimisation of the photolysis of aryl azide **8a**.

Aryl azide **8a** bearing a *p*-methyl ester substituent, prepared by treatment of the corresponding aniline with NaNO_2_/HCl–NaN_3_, was selected for initial optimization of the reaction (Scheme 2). During preliminary experiments under the conditions reported by Smalley (0.05 M in THF/H_2_O, 1:1) [31], blockage of the tubing was observed, which was attributed to the poor solubility of the substrate in water. Running the reaction with a reduced water content (THF/H_2_O, 4:3) obviated this problem and resulted in a 62% conversion of the starting azide to 3*H*-azepinone **9a**, after a residence time of 30 min (Table 1, entry 2). Further reduction in the water content had a detrimental effect on the conversion. An inverse dependence of the conversion on substrate concentration was observed (Table 1). Although the highest conversion was observed at 0.015 M, higher productivity was obtained at 0.030 M (0.38 mmol h^−1^). Representing the best balance between conversion and productivity, 0.030 M was selected as the optimum concentration for further optimization. The use of other polar, aprotic, water-miscible solvents for the reaction was explored. Maintaining a residence time of 30 min, no significant change in conversion was observed upon replacing THF with DME or 1,4-dioxane; decreased conversion (22%) was observed with acetonitrile as the organic solvent.

**Table 1:** Effect of aryl azide concentration on conversion.

Entry	[**8a**] (M)	Conversion (%)^a^	Isolated Yield (%)

1	0.100	39	29
2	0.050	62	35
3	0.030	78	45
4	0.015	90	48

^a^By ^1^H NMR analysis of the crude product mixture after a residence time of 30 min.

**Figure 1:**
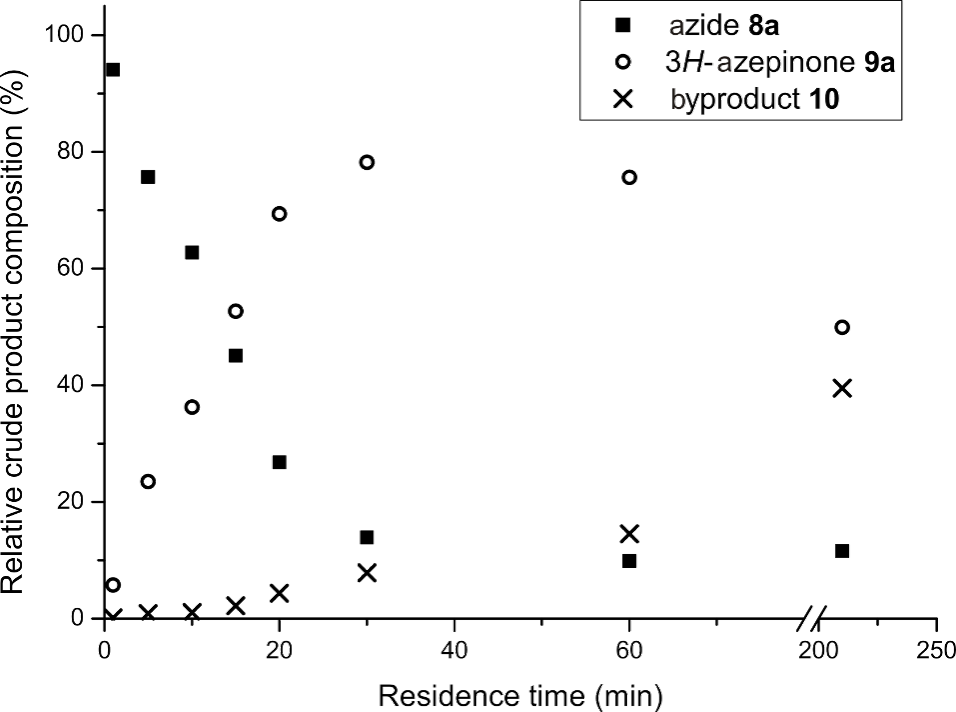
Relationship between residence time and relative composition of the crude reaction mixture.

**Scheme 3:**
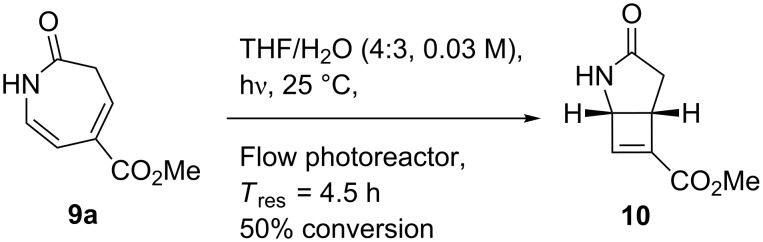
Preparation of side product **10**.

Increasing the residence time improved the conversion of **8a** (Figure 1), although this was accompanied by increased concomitant formation of the byproduct **10**. Lactam **10** was obtained upon re-exposure of purified **9a** to the reaction conditions and is believed to result from photochemical disrotatory electrocyclization of the 3*H*-azepinone diene moiety [51–54] (Scheme 3). Instead of making a direct comparison between reaction progress in batch and flow, the effects of longer residence times reported for batch photolysis were evaluated in a stopped-flow experiment by irradiating a solution of **8a** for 3.5 h in the same FEP photoreactor (Figure 1). Analysis of the composition of the crude mixture showed significantly increased conversion of **9a** to **10** accompanied by a slight increase in the proportion of the starting material **8a**, which likely reflects decomposition of both of the reaction products. Overall, a residence time of 30 min was selected for a 0.030 M solution of **8a** in THF/H_2_O 4:3, giving analytically pure **9a** in 45% yield. Only traces of the diazo product were observed in the NMR spectrum of the crude mixture.

**Scheme 4:**
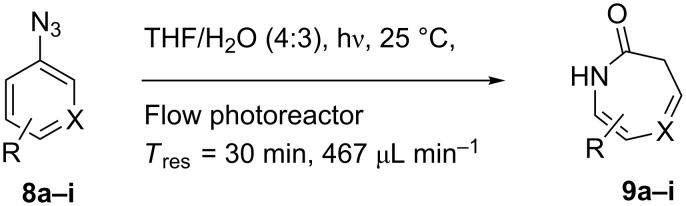
General conditions for the photolysis of aryl azides in continuous flow.

A variety of other 3*H*-azepinones was prepared in moderate to good yields under the optimized reaction conditions (Scheme 4, Table 2). Residual starting material was easily recovered by silica column chromatography. Rearrangements of aryl azides bearing electron withdrawing substituents are better represented in the literature than their electron donating congeners [31], and this was reflected in our compound selection. Methyl 2-azidobenzoate (**8b**), the corresponding acid **8c** and dimethyl 2-azidoterephthalate (**8d**) each reacted favorably compared to the model substrate **8a**. We were particularly interested in the preparation of 5-aryl-3*H*-azepinones **9e–g**, examples of which have seen recent interest as γ-secretase inhibitors for the treatment of Alzheimer’s disease [37]. Photolysis of methyl 2-azido-5-phenylbenzoate **8e** under the optimized conditions afforded a complex mixture of products. Upon shortening the residence time to 15 min, 3-methoxycarbonyl-5-phenyl-3*H*-azepinone (**9e**) was obtained in 35% yield. While both the corresponding acid **8f** and 4-azidobiphenyl **8g** decomposed upon photolysis, **8h** gave the corresponding 5-chloro-3*H*-azepinone **9h**, bearing a handle for further functionalization. To explore the preparation of bicyclic products, we studied the photolysis of 3-azidoquinoline (**8i**) to give the corresponding benzodiazepinone. Although the substrate decomposed under the original optimized conditions, reaction of **8i** in NaOMe–MeOH [55–56] afforded **9i** in good yield. Although yields were similar to those reported for batch processes [31,34,56], performing the reaction in continuous flow allows simple scaleup.

**Table 2:** 3*H*-azepinone derivatives prepared by photolysis of aryl azides in continuous flow.

Entry	Substrate		Product		Yield (%)^a^

1	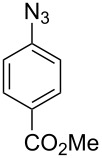	**8a**	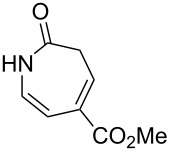	**9a**	45 (51)
2	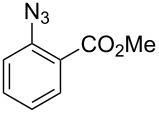	**8b**	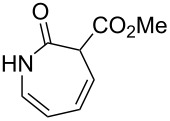	**9b**	75 (77)
3	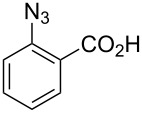	**8c**	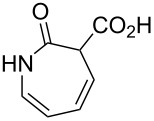	**9c**	50 (62)
4	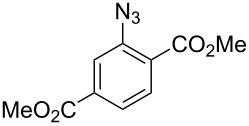	**8d**	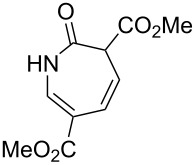	**9d**	74 (80)
5^b^	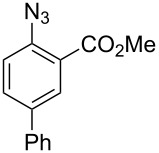	**8e**	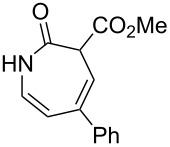	**9e**	35 (47)
6	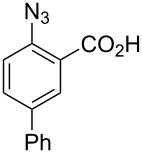	**8f**	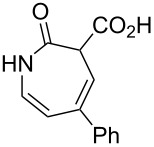	**9f**	0
7	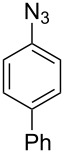	**8g**	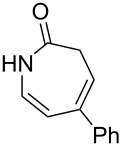	**9g**	0
8	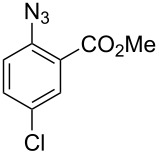	**8h**	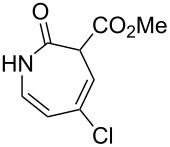	**9h**	70
9^c^	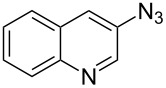	**8i**	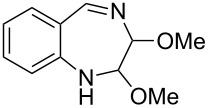	**9i**	44^d^

^a^Yields in parentheses are based on recovered starting material. ^b^The residence time was reduced to 15 min. ^c^The photolysis was performed with a 0.030 M solution of **8i** in 1.0 M NaOMe–MeOH. ^d^Determined by ^1^H NMR of the crude mixture.

In summary, the photochemical syntheses of 3*H*-azepinones and related azepines were performed and optimized in continuous flow through a photoreactor made from FEP tubing. Secondary photochemical reactions were minimized through careful control of the residence time. Although isolated yields were variable, a variety of aryl azides bearing electron withdrawing substituents underwent photolysis in good yield. Modification of the procedure allowed the preparation of benzodiazepine **9i**.

## Supporting Information

File 1 Description of the flow reactor setup, experimental procedures and spectroscopic data of all compounds.
