# Supplementary material for: Continuous flow photolysis of aryl azides: Preparation of 3H-azepinones
Source: Beilstein J Org Chem. 2011 Aug 17;7:1124–9. doi: 10.3762/bjoc.7.129 (PMC3167900; doi:10.3762/bjoc.7.129)
Supplement: File 1 — Description of the flow reactor setup, experimental procedures and spectroscopic data of all compounds. [file Beilstein_J_Org_Chem-07-1124-s001.pdf]

Supporting Information
for

Continuous flow photolysis of aryl azides: Preparation of 3*H*-azepinones.

Farhan R. Bou-Hamdan‡, François Lévesque‡, Alexander G. O'Brien‡ and Peter. H. Seeberger*

Address: Max Planck Institute of Colloids and Interfaces, Department of Biomolecular Systems, Am Mühlenberg 1, 14476 Potsdam, Germany; and Freie Universität Berlin, Institute for Chemistry and Biochemistry, Arnimallee 22, 14195 Berlin, Germany.

Email: Peter H. Seeberger - peter.seeberger@mpikg.mpg.de

*Corresponding author

‡These authors contributed equally to the work

Description of the flow reactor setup, experimental procedures and spectroscopic data of all compounds

1. General information (S2)
2. Flow reactor setup (S3)
3. Procedures and spectroscopic data (S5)
4. ^{1}H and ^{13}C NMR data of all compounds (S17)

1. General information

All chemicals were reagent grade and used as supplied, except where noted. THF for photolysis was HPLC grade. The term “concentrated under reduced pressure” refers to the removal of solvents and other volatile material using a rotary evaporator while maintaining a water bath temperature under 40 °C. The compounds purified over silica gel were further concentrated by the removal of residual solvent under high vacuum (<0.2 mbar).

^{1}H NMR spectra were recorded on a Varian 400-MR (400 MHz) spectrometer at ambient temperature. The proton signal of residual non-deuterated solvent (δ 7.26 ppm for $CHCl_3$, δ 2.50 ppm for DMSO, δ 2.05 ppm for acetone or δ 4.79 ppm for H_2O) was used as an internal reference for ^{1}H spectra. Data are reported as follows: chemical shift in parts per million (δ, ppm), multiplicity (s = singlet, bs = broad singlet, d = doublet, t = triplet, q = quartet, qn = quintet and m = multiplet), coupling constants reported in Hertz (Hz) and integration. ^{13}C spectra were recorded on a Varian VXR-400 spectrometer (at 100 MHz) at ambient temperature. Chemical shifts are reported in parts per million (δ, ppm). The carbon signal of deuterated solvent (δ 77.16 ppm for $CDCl_3$, δ 29.84 ppm for d_6-DMSO or δ 39.52 ppm for d_6-acetone) was used as an internal reference for ^{13}C spectra. Infrared (IR) spectra were recorded as thin films on a Perkin-Elmer 1600 FTIR spectrophotometer. High-resolution mass spectra (HRMS) were recorded with an Agilent 6210 ESI-TOF mass spectrometer at the Freie Universität Berlin, Mass Spectrometry Core Facility. Analytical thin layer chromatography (TLC) was performed on Kieselgel 60 F254 glass plates precoated with a 0.25 mm thickness of silica gel. The TLC plates were visualized with UV light and by staining with Hanessian solution (ceric sulfate and ammonium molybdate in aqueous sulfuric acid), basic $KMNO_4$ or vanillin. Column chromatography was performed using Kieselgel 60 (230–400 mesh) silica gel with a typical 50-100: 1 weight ratio of silica gel to crude product. Melting points were recorded using a Thermo Fisher IA 9300 melting point apparatus and are uncorrected.

2. Continuous flow reactor setup.

The flow reactor setup consisted of a Vapourtec R2+ pumping module (**1**), [1] a PTFE T-mixer, multiple loops of FEP tubing (**2**, fluorinated ethylene polymer from IDEX Health & Science 1520, natural color, outside diameter (OD) 1/16 in and inside diameter (ID) 0.030 in) [2] wrapped tightly around a Pyrex filter (**3**, inner diameter 4.5 cm and wall thickness 0.2 cm), surrounding the quartz immersion well (**4**) cooled by a recirculating cryostat (Huber Unistat 360), a medium pressure Hg lamp (**5**, UV 450 immersion lamp 5 in. arc, radial lead, 7825-34 from Ace Glass), [3] a power supply for photochemical lamp (7830 from Ace Glass), a back-pressure regulator of 6.9 bar (**6**, U-607 from IDEX) [2] and a collection flask (Figure SI-4). The inlet valve of the R2+ pump system (Vapourtec) was used for rapid switching from pure solvent to the solution containing the dissolved starting material at the intake pump.

FEP tubing was selected for its high transmittance and stability in the UV–vis light range, [4] its flexibility and its high chemical resistance. The 2 mm thick Pyrex filter was essential to absorb wavelengths below 300 nm, to prevent degradation of the tubing, and to avoid any undesired side reactions caused by short wavelength radiation. The temperature in the tube during the reaction is estimated to range from 25 to 30 °C, based on temperature measurements taken between the cooling jacket and the tube. For safety reasons, the lamp was placed inside an aluminium box to block UV irradiation. Two fans were installed for additional cooling.

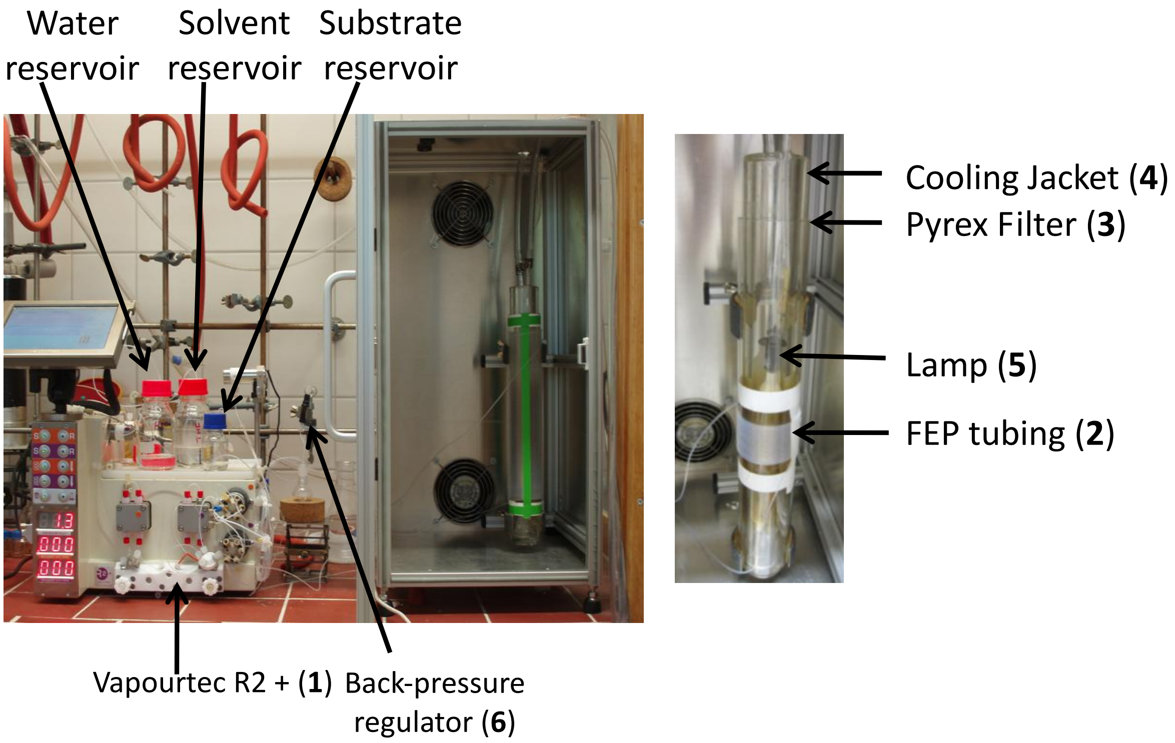

Figure SI-1: Picture of the flow reactor setup.

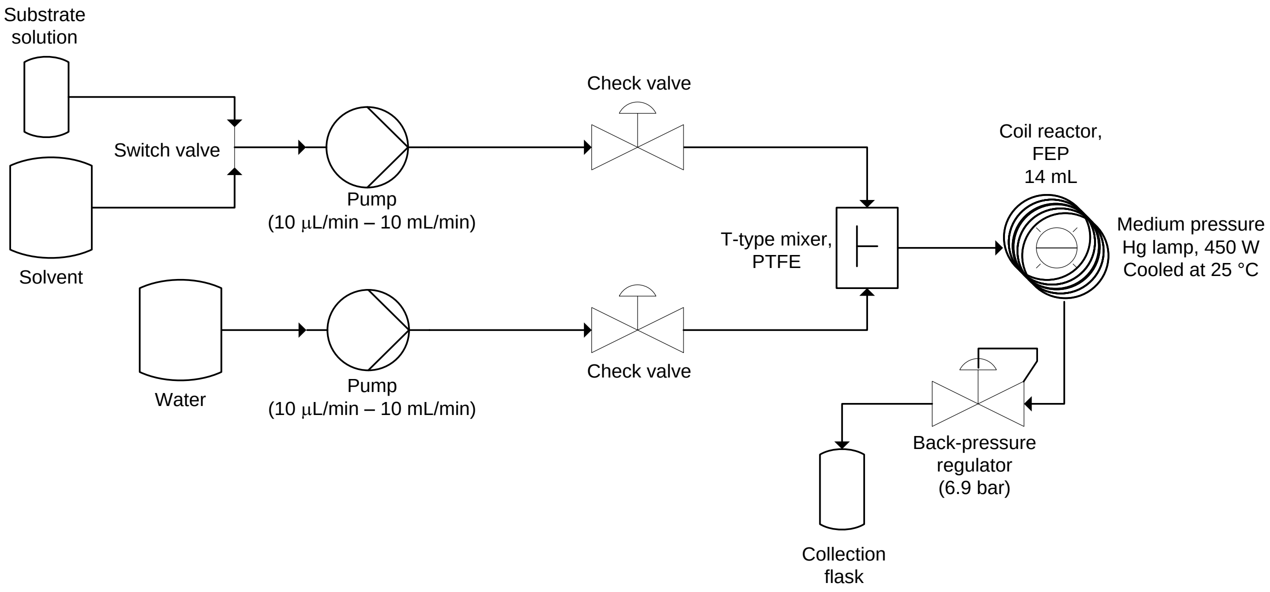

Figure SI-2: System diagram of the flow reactor setup.

3. Experimental procedures and spectroscopic data

Procedures for the preparation of aryl azides [5]

Methyl 4-azidobenzoate (8a)

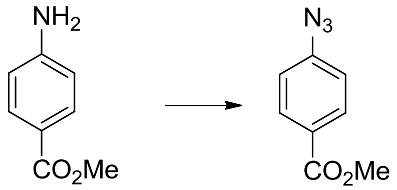

To a solution of methyl 4-aminobenzoate (3.25 g, 21.5 mmol) in aqueous hydrochloric acid (3 M, 120 mL) at 0 °C was added a solution of sodium nitrite (2.82 g, 40.9 mmol, 1.9 equiv) in water (25 mL) dropwise, while maintaining a temperature below 5 °C. After stirring for 1 h at 0 °C, a solution of sodium azide (2.80 g, 43.0 mmol, 2.0 equiv) in water (50 mL) was added dropwise while maintaining the same low temperature, and the mixture was stirred for an additional 1 h at 0 °C. The resulting suspension was filtered, the residual solid was washed thoroughly with water and dried in the dark under reduced pressure to afford methyl 4-azidobenzoate (**8a**, 3.57 g, 94%) as a light brown solid. ^{1}H NMR (400 MHz, $CDCl_3$) δ 8.03 (d, J = 8.5 Hz, 2H), 7.06 (d, J = 8.5 Hz, 2H), 3.91 (s, 3H); ^{13}C NMR (100 MHz, $CDCl_3$) δ 166.3, 144.7, 131.4, 126.7, 118.8, 52.1; in agreement with published data. [6]

Methyl 2-azidobenzoate (8b)

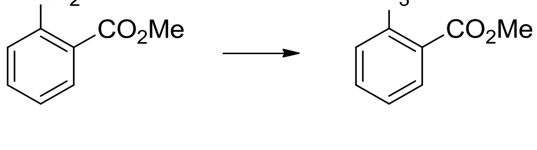

To a solution of methyl 2-aminobenzoate (1.59 g, 10.5 mmol) in aqueous hydrochloric acid (3 M, 50 mL) at 0 °C was added a solution of sodium nitrite (1.38 g, 20.0 mmol, 1.9 equiv) in water (10 mL) dropwise, while maintaining a temperature below 5 °C. After stirring for 1 h at 0 °C, a solution of sodium azide (1.37 g, 21.0 mmol, 2.0 equiv) in water (20 mL) was added dropwise while maintaining the same low temperature, and the mixture was stirred for an additional 1 h at 0 °C. The resulting emulsion was extracted

with EtOAc (3×), and the combined organic phases were washed with water (1×), saturated aqueous $NaHCO_3$ (2×), brine (1×) and dried (Na_2SO_4). Removal of the solvent under reduced pressure afforded methyl 2-azidobenzoate (**8b**, 0.95 g, 51%) as a dark red oil. ^{1}H NMR (400 MHz, $CDCl_3$) δ 7.86–7.81 (m, 1H), 7.54–7.47 (m, 1H), 7.24–7.19 (m, 1H), 7.18–7.12 (m, 1H), 3.89 (s, 3H); ^{13}C NMR (100 MHz, $CDCl_3$) δ 165.6, 139.9, 133.1, 131.7, 124.4, 122.5, 119.8, 52.2; in agreement with published data. [7]

2-Azidobenzoic acid (8c)

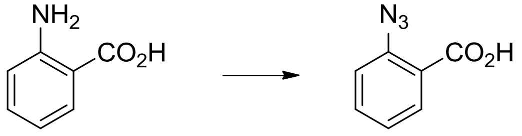

To a solution of anthranilic acid (3.25 g, 23.7 mmol) in aqueous hydrochloric acid (3 M, 125 mL) at 0 °C was added a solution of sodium nitrite (3.11 g, 45.0 mmol, 1.9 equiv) in water (25 mL) dropwise, while maintaining a temperature below 5 °C. After stirring for 1 h at 0 °C, a solution of sodium azide (3.08 g, 47.4 mmol, 2.0 equiv) in water (50 mL) was added dropwise while maintaining the same low temperature. The mixture was stirred for an additional 2 h at 0 °C, then allowed to warm up to rt overnight. The resulting suspension was filtered, and the residual solid was washed thoroughly with water, dissolved in EtOAc, dried (Na_2SO_4) and concentrated under reduced pressure to afford 2-azidobenzoic acid (**8c**, 3.03 g, 78%) as a light brown solid. ^{1}H NMR (400 MHz, d_6-DMSO) δ 13.13 (bs, 1H), 7.77 (dd, J = 8.0, 1.5 Hz, 1H), 7.60 (td, J = 8.0, 1.5 Hz, 1H), 7.36 (d, J = 8.0 Hz, 1H), 7.27 (td, J = 8.0, 1.0 Hz, 1H); ^{13}C NMR (100 MHz, d_6-DMSO) δ 166.4, 138.7, 133.0, 131.1, 124.9, 124.0, 120.8; in agreement with published data. [8]

Dimethyl azidoterephthalate (8d)

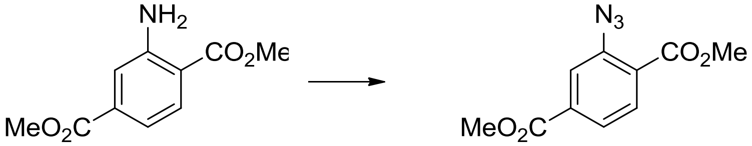

To a solution of dimethyl aminoterephthalate (2.50 g, 12.0 mmol) in aqueous hydrochloric acid (3 M, 110 mL) at 0 °C was added a solution of sodium nitrite (1.58 g, 22.7 mmol, 1.9 equiv) in water (43 mL)

dropwise, while maintaining a temperature below 5 °C. After stirring for 1 h at 0 °C, a solution of sodium azide (1.55 g, 23.9 mmol, 2.0 equiv) in water (38 mL) was added dropwise while maintaining the same low temperature and the mixture was stirred for an additional 2 h at 0 °C. The resulting suspension was filtered, and the residual solid was washed thoroughly with water and dried. The solid was taken up in EtOAc, dried (Na_2SO_4), filtered and concentrated under reduce pressure to afford dimethyl azidoterephthalate (**8d**, 2.61 g, 93%) as a brown solid. ^{1}H NMR (400 MHz, $CDCl_3$) δ 7.87–7.85 (m, 2H), 7.79 (dd, *J* = 8.0, 1.5 Hz, 1H), 3.94 (s, 3H), 3.91 (s, 3H); ^{13}C NMR (100 MHz, $CDCl_3$) δ 165.4, 165.3, 140.4, 134.4, 131.9, 126.3, 125.3, 120.9, 52.8, 52.7; IR-thin film (ν, cm^{-1}) 3022, 2966, 2155, 2122, 1717, 1489, 1434, 1398, 1305, 1292, 1248; in agreement with published data. [9]

Methyl 2-azido-5-phenylbenzoate (8e)

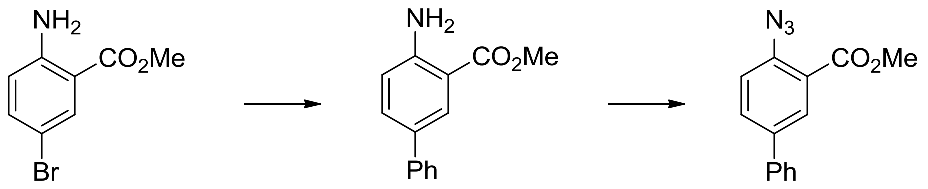

To a solution of methyl 2-amino-5-bromobenzoate (5.0 g, 21.5 mmol) and $Pd(Ph_3P)_4$ (0.500 g, 0.435 mmol, 0.02 equiv) in toluene (50 mL) was added a solution of Na_2CO_3 (2.53 g, 23.9 mmol, 1.1 equiv) in water (24 mL). After stirring for 5 min at rt, a solution of phenylboronic acid (3.58 g, 29.3 mmol, 1.35 equiv) in MeOH (13 mL) was added, and the mixture was stirred at rt for an additional 10 min. The reaction mixture was heated at reflux for 4 h 45 min then cooled to rt. Water was added and the mixture was filtered over a pad of Celite®, and the residue was washed with toluene. The phases were separated and the aqueous phase was extracted with EtOAc (2×). The combined organic phases were washed with saturated aqueous $NaHCO_3$ (3×), brine, dried (Na_2SO_4) and concentrated under reduced pressure to give methyl 2-amino-5-phenylbenzoate (3.72 g, 75%) as an off-white solid following purification over silica gel (20–34% EtOAc/hexanes). ^{1}H NMR (400 MHz, $CDCl_3$) δ 8.14 (d, *J* = 2.5 Hz, 1H), 7.59–7.51 (m, 3H), 7.45–7.37 (m, 2H), 7.33–7.27 (m, 1H), 6.75 (d, *J* = 8.5 Hz, 1H), 5.79 (bs, 2H), 3.91 (s, 3H); ^{13}C NMR (100 MHz, $CDCl_3$) δ 168.5, 149.7, 140.4, 132.8, 129.5, 129.3, 128.7, 126.4, 126.2, 117.2, 110.9, 51.6; in agreement with published data. [10]

To a solution of methyl 2-amino-5-phenylbenzoate (1.50 g, 6.6 mmol) in aqueous hydrochloric acid (3 M, 60 mL) at 0 ºC was added a solution of sodium nitrite (0.87 g, 12.5 mmol, 1.9 equiv) in water (24 mL) dropwise, while maintaining a temperature below 5°C. After stirring for 1 h at 0 °C, a solution of sodium azide (0.86 g, 13.2 mmol, 2.0 equiv) in water (21 mL) was added dropwise while maintaining the same low temperature, and the mixture was stirred for an additional 2 h at 0 °C. The resulting suspension was filtered, the residual solid was washed thoroughly with water and dried. The product was taken up in EtOAc, dried (Na_2SO_4) and concentrated under reduce pressure to afford methyl 2-azido-5-phenylbenzoate (**8e**, 1.60 g, 95%) as a brown solid. ^{1}H NMR (400 MHz, $CDCl_3$) δ 8.10 (d, *J* = 2.5 Hz, 1H), 7.75 (dd, *J* = 8.5, 2.5 Hz, 1H), 7.59–7.56 (m, 2H), 7.48–7.43 (m, 2H), 7.38 (ddt, *J* = 8.5, 6.5, 1.5 Hz, 1H), 7.32 (d, *J* = 8.5 Hz, 1H), 3.94 (s, 3H); ^{13}C NMR (100 MHz, $CDCl_3$) δ 165.9, 139.2, 139.1, 137.8, 131.7, 130.5, 129.1, 128.0, 127.0, 123.0, 120.5, 52.6; IR-thin film (ν, cm^{-1}) 3032, 2951, 2118, 2092, 1725, 1418, 1451, 1434, 1300, 1225; HRMS–ESI: M+Na, calc. 276.0749, meas. 276.0750.

2-Azido-5-phenylbenzoic acid (8f)

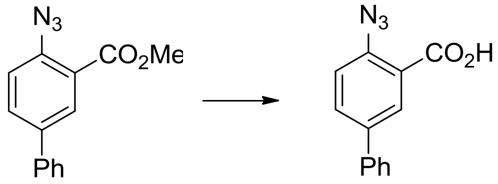

To a solution of methyl 2-azido-5-phenylbenzoate (**8e**, 0.50 g, 2.0 mmol) in THF (8.2 mL) and MeOH (2.5 mL) was added a solution of lithium hydroxide (0.057 g, 2.4 mmol, 1.2 equiv) in water (2.5 mL) dropwise. After stirring for 2 h at rt, the solution was acidified with aqueous hydrochloric acid (1 M). EtOAc was added and the two phases were separated. The aqueous phase was extracted with EtOAc (2×). The combined organic phases were dried (Na_2SO_4), filtered and concentrated under reduce pressure to afford 2-azido-5-phenylbenzoic acid (**8f**, 0.47 g, >99%) as a brown solid. ^{1}H NMR (400 MHz, $CDCl_3$) δ 8.41 (d, *J* = 2.5 Hz, 1H), 7.85 (dd, *J* = 8.5, 2.5 Hz, 1H), 7.62–7.59 (m, 2H), 7.49–7.45 (m, 2H), 7.40 (ddt, *J* = 8.5, 6.5, 1.5 Hz, 1H), 7.35 (d, *J* = 8.5 Hz, 1H); ^{13}C NMR (100 MHz, $CDCl_3$) δ 167.1, 138.8, 138.7, 138.5, 132.9, 132.0, 129.2, 128.2, 127.0, 121.0, 120.0; IR-thin film (ν, cm^{-1}) 2870 (br), 2120, 2082, 1700, 1479, 1418, 1304, 1251; HRMS–ESI: M+Na, calc. 262.0592, meas. 262.0602.

4-Azidobiphenyl (8g)

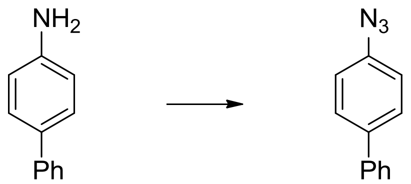

To a solution of 4-aminobiphenyl (2.50 g, 14.8 mmol) in aqueous hydrochloric acid (3 M, 60 mL) at 0 ºC was added a solution of sodium nitrite (1.94 g, 28.1 mmol, 1.9 equiv) in water (24 mL) dropwise, while maintaining a temperature below 5 °C. Additional DMF (5 mL) was added. After stirring for 1 h at 0 °C, a solution of sodium azide (1.92 g, 29.5 mmol, 2.0 equiv) in water (20 mL) was added dropwise while maintaining the same low temperature, and the mixture was stirred for an additional 1.5 h at 0 °C. The resulting suspension was filtered, and the residual solid was washed thoroughly with water and dried in air. The solid was taken up in EtOAc, dried (Na_2SO_4), filtered and concentrated under reduced pressure to afford 4-azidobiphenyl (**8g**, 2.72 g, 94%) as an orange solid. ^{1}H NMR (400 MHz, $CDCl_3$) δ 7.61–7.56 (m, 4 H), 7.47-7.43 (m, 2 H), 7.36 (ddt, *J* = 8.0, 6.5, 1.5 Hz, 1 H), 7.13–7.09 (m, 2 H); ^{13}C NMR (100 MHz, $CDCl_3$) δ 140.3, 139.3, 138.1, 129.0, 128.6, 127.5, 127.0, 119.5; in agreement with published data. [11]

Methyl 2-azido-5-chlorobenzoate (8h)

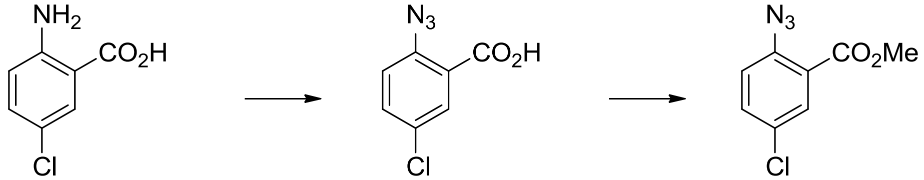

To a solution of 2-amino-5-chlorobenzoic acid (2.00 g, 17.5 mmol) in aqueous hydrochloric acid (3 M, 160 mL) at 0 ºC was added a solution of sodium nitrite (2.29 g, 33.2 mmol, 1.9 equiv) in water (63 mL) dropwise, while maintaining a temperature below 5 °C. After stirring for 1 h at 0 °C, a solution of sodium azide (2.27 g, 35.0 mmol, 2.0 equiv) in water (55 mL) was added dropwise while maintaining the same low temperature, and the mixture was allowed to warm to rt overnight. The resulting suspension was filtered, and the residual solid was washed thoroughly with water and dried in air. The solid was taken up in EtOAc, dried (Na_2SO_4), and concentrated under reduced pressure to afford 2-azido-5-chlorobenzoic acid (2.97 g,

86%) as an off-white solid. ^{1}H NMR (400 MHz, $CDCl_3$) δ 8.12 (d, *J* = 2.5 Hz, 1H), 7.57 (dd, *J* = 8.5, 2.5 Hz, 1H), 7.22 (d, *J* = 8.5 Hz, 1H); ^{13}C NMR (100 MHz, $CDCl_3$) δ 166.6, 138.8, 134.4, 133.2, 130.8, 122.1, 121.0; IR-thin film (ν, cm^{-1}) 3090, 2149, 2123, 1720, 1679, 1592, 1480, 1419, 1300, 1253; HRMS–ESI: M–H, calc. 195.9914, meas. 195.9944; in agreement with published data. [7]

To a solution of 2-azido-5-chlorobenzoic acid (0.69 g, 3.49 mmol) and *N*-methylmorphiline (0.46 mL, 4.19 mmol, 1.2 equiv) in toluene (18 mL) at 0 ºC was added ethyl chloroformate (0.40 mL, 4.19 mmol, 1.2 equiv) maintaining a temperature below 5 °C. After stirring for 1 h at 0 °C, MeOH (2.8 mL, 70 mmol, 20 equiv) and 4-*N*,*N*-dimethylaminopyridine (85 mg, 0.70 mmol, 0.2 equiv) were added. The resulting solution was stirred at rt for 14 h. Additional MeOH (10 mL, 250 mmol, 71 equiv) was added and the resulting solution was heated to 70 °C. After 1 h, the reaction was cooled to rt and concentrated under reduced pressure. The product was taken up in EtOAc and washed with aqueous hydrochloric acid (1 M). The aqueous phase was further extracted with EtOAc. The combined organic extracts were washed with saturated aqueous $NaHCO_3$ (2×), brine, dried (Na_2SO_4) and concentrated under reduced pressure to give methyl 2-azido-5-chlorobenzoate **8h** (0.41 g, 56%) as an off-white solid. ^{1}H NMR (400 MHz, $CDCl_3$) δ 7.84 (d, *J* = 2.5 Hz, 1H), 7.48 (dd, *J* = 8.5, 2.5 Hz, 1H), 7.17 (d, *J* = 8.5 Hz, 1H), 3.91 (s, 3H); ^{13}C NMR (100 MHz, $CDCl_3$) δ 164.6, 138.8, 133.2, 131.8, 130.0, 123.8, 121.4, 52.7; IR-thin film (ν, cm^{-1}) 3104, 3086, 2954, 2926, 2125, 2090, 1728, 1485, 1436, 1300, 1247; HRMS – ESI: M+Na, calc. 234.0046, meas. 234.0057.

3-Azidoquinoline (8i)

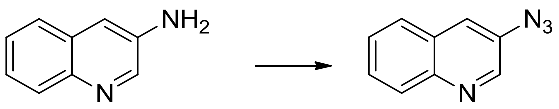

To a solution of 3-aminoquinoline (2.00 g, 13.9 mmol) in aqueous hydrochloric acid (3 M, 20 mL) at 0 ºC was added a solution of sodium nitrite (1.15 g, 16.7 mmol, 1.2 equiv) in water (15 mL) dropwise, while maintaining a temperature below 5 °C. After stirring for 1 h at 0 °C, a solution of sodium azide (1.80 g, 27.7 mmol, 2.0 equiv) in water (10 mL) was added dropwise while maintaining the same low temperature, and the mixture was stirred for an additional 1 h at 0 °C. The resulting mixture was neutralised with a

saturated aqueous solution of $NaHCO_3$ and extracted with DCM (3×). The combined organic phases were washed with brine, dried (Na_2SO_4) filtered and concentrated under reduce pressure to afford 3-azidoquinoline (**8i**, 2.33 g, 99%) as a yellow solid. ^{1}H NMR (400 MHz, $CDCl_3$) δ 8.62 (d, *J* = 3.0 Hz, 1H), 8.08 (dd, *J* = 8.5, 0.5 Hz, 1H), 7.81–7.72 (m, 2H), 7.66 (ddd, *J* = 8.5, 7.0, 1.5 Hz, 1H), 7.56 (ddd, *J* = 8.0, 7.0, 1.5 Hz, 1H); ^{13}C NMR (100 MHz, $CDCl_3$) δ 145.8, 143.8, 133.9, 129.5, 128.7, 128.1, 127.7, 126.8, 122.5; in agreement with published data. [12]

General Procedure for Preparation of 3*H*-azepinones

The UV lamp was turned on 30 min prior to use. A Vapourtec R2 series flow reactor system connected to a FEP photoreactor was set up as described above. THF and H_2O were injected simultaneously into the reactor at the specified rates, and mixed by a PTFE T-mixer. After stabilization of the system, the intake of one of the pumps was switched to a solution containing the arylazide **8** in THF. The entire reaction mixture was collected and extracted with EtOAc (3X). The combined organic phases were washed with brine (2X), dried (Na_2SO_4), filtered and concentrated under reduced pressure. The conversion of each run was determined by ^{1}H NMR of the crude product, and the desired 3*H*-azepin-2-one products were purified by flash chromatography.

5-methoxycarbonyl-3*H*-azepin-2-one (9a)

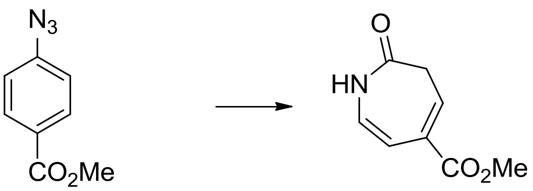

According to the general procedure, a solution of methyl 4-azidobenzoate (**8a**, 0.232 g, 1.31 mmol) in THF (25.8 mL) was injected at a rate of 0.267 mL/min, mixing with water at a rate of 0.200 mL/min (30 min residence time). The crude product (80% conversion by ^{1}H NMR), was purified over silica gel (40–60% EtOAc/hexanes) to afford the recovered starting material **8a** (30 mg, 13%) and 5-methoxycarbonyl-3*H*-azepin-2-one (**9a**, 0.097 g, 45%, 51% *brsm*) as a yellow solid: mp 110–111 ºC (lit 112 ºC); ^{1}H NMR (400 MHz, $CDCl_3$) δ 8.12 (bs, 1H), 6.71 (t, *J* = 7.5 Hz, 1H), 6.34–6.27 (m, 2H), 3.79 (s, 3H), 3.03 (d, *J* = 7.5 Hz,

2H); ^{13}C NMR (100 MHz, $CDCl_3$) δ 168.0, 165.8, 131.6, 129.8, 126.7, 111.8, 52.2, 37.2; IR-thin film (ν, cm^{-1}) 3202, 3093, 2956, 1725, 1667, 1597, 1437, 1367, 1254, 1176; HRMS–ESI: M+Na, calc. 190.0480, meas. 190.0466; in agreement with published data. [9]

3-Methoxycarbonyl-3*H*-azepin-2-one (9b)

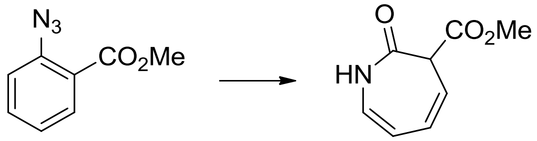

According to the general procedure, a solution of methyl 2-azidobenzoate (**8b**, 0.232 g, 1.31 mmol) in THF (25.8 mL) was injected at a rate of 0.267 mL/min, mixing with water at a rate of 0.200 mL/min (30 min residence time). The crude product (91% conversion by ^{1}H NMR), was purified by over silica gel (50–60% EtOAc/hexanes) to afford the recovered starting material **8b** (5 mg, 2%) and 3-methoxycarbonyl-3*H*-azepin-2-one (**9b**, 0.165 g, 75%, 77% *brsm*) as a yellow solid: m.p. 109–111 ºC; ^{1}H NMR (400 MHz, $CDCl_3$) δ 8.24 (bs, 1H), 6.27 (ddd, J = 9.5, 5.0, 1.5 Hz, 1H), 6.23 (ddd, J = 9.0, 8.0, 0.5 Hz, 1H), 5.95 (dd, J = 9.5, 6.0 Hz, 1H), 5.91 (dd, J = 9.0, 5.0 Hz, 1H), 3.85 (s, 3H), 3.54 (ddd, J = 6.0, 2.0, 0.5 Hz, 1H); ^{13}C NMR (100 MHz, $CDCl_3$) δ 168.8, 164.5, 126.6, 125.7, 120.7, 114.5, 52.7, 52.5; IR-thin film (ν, cm^{-1}) 3247, 3116, 2956, 1742, 1662, 1634, 1593, 1321, 1236, 1212, 1197, 1157; HRMS–ESI: M+Na, calc. 190.0480, meas. 190.0469; in agreement with published data. [7]

3*H*-Azepin-2-one-3-carboxylic acid (9c)

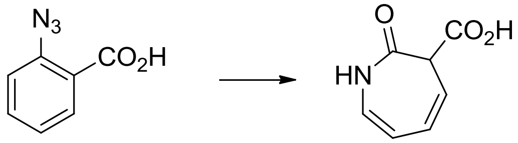

According to the general procedure, a solution of 2-azidobenzoic acid (**8c**, 0.214 g, 1.31 mmol) in THF (25.8 mL) was injected at a rate of 0.267 mL/min, mixing with water at a rate of 0.200 mL/min (30 min residence time). The crude product was purified over silica gel (0–5% AcOH/EtOAc) to afford the recovered starting material **8c** (43 mg, 20%) and 3*H*-azepin-2-one-3-carboxylic acid (**9c**, 0.100 g, 50%,

62% *brsm*) as an oily yellow solid: ^{1}H NMR (400 MHz, CD_3OD) δ 6.38– 6.20 (m, 2H), 5.95 (dd, *J* = 9.0, 5.5 Hz, 1H), 5.86 (dd, *J* = 9.5, 6.0 Hz, 1H), 3.45 (dd, *J* = 6.0, 1.5 Hz, 1H); ^{13}C NMR (100 MHz, CD_3OD) δ 171.9, 166.0, 127.8, 127.0, 122.2, 115.3, 54.0; IR-thin film (ν, cm^{-1}) 3449 (br), 3320, 3114, 2956, 2636 (br), 1724, 1656, 1633, 1590, 1416, 1331, 1239, 1197, 1095, 1041; HRMS–ESI: M+Na, calc. 176.0324, meas. 176.0315; in agreement with published data. [9

3,6-Bis(methoxycarbonyl)-3*H*-azepin-2-one (9d)

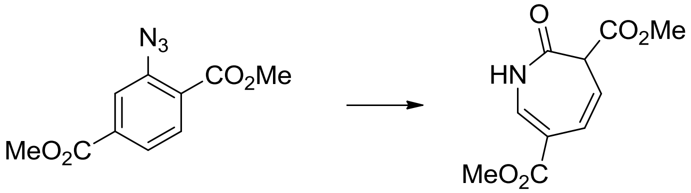

According to the general procedure, a solution of dimethyl azidoterephthalate (**8d**, 0.308 g, 1.31 mmol) in THF (25.8 mL) was injected at a rate of 0.267 mL/min, mixing with water at a rate of 0.200 mL/min (30 min residence time). The crude product was purified over silica gel (10–50% EtOAc/hexanes) to afford the recovered starting material **8d** (25 mg, 8%) and 3,6-bis(methoxycarbonyl)-3*H*-azepin-2-one (**9d**, 0.218 g, 74%, 80% *brsm*) as a yellow solid: ^{1}H NMR (400 MHz, $CDCl_3$) δ 8.64 (bs, 1H), 7.40 (d, *J* = 6.0 Hz, 1H), 6.76 (dd, *J* = 10.0, 2.0 Hz, 1H), 6.01 (d, *J* = 10.0, 6.0 Hz, 1H), 3.85 (s, 3H), 3.81 (s, 3H), 3.55 (dd, *J* = 6.0, 2.0 Hz, 1H); ^{13}C NMR (100 MHz, $CDCl_3$) δ 168.0, 166.5, 164.0, 133.7, 125.7, 120.4, 117.8, 53.02, 53.00, 52.4; IR-thin film (ν, cm^{-1}) 3537, 3269, 2956, 1749, 1695, 1635, 1599, 1438, 1273, 1223, 1201; HRMS–ESI: M+Na, calc. 248.0535, meas. 248.0542; in agreement with published data. [9]

3-Methoxycarbonyl-5-phenyl-3*H*-azepin-2-one (9e)

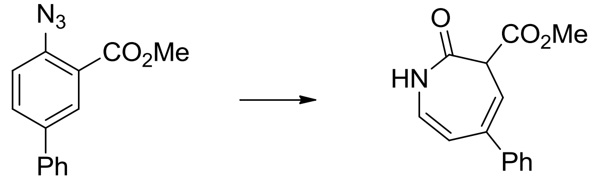

According to the general procedure, a solution of solution of methyl 2-azido-5-phenylbenzoate (**8e**, 0.343 g, 1.36 mmol) in THF (25.8 mL) was injected at a rate of 0.534 mL/min, mixing with water at a rate of 0.400 mL/min (15 min residence time). The crude product was purified over silica gel (10–50% EtOAc/hexanes) to afford the recovered starting material **8e** (86 mg, 25%) and 3-methoxycarbonyl-5-phenyl-3*H*-azepin-2-one (**9e**, 0.115 g, 35%, 47% *brsm*) as a yellow solid: m.p. 121–124 ºC. ^{1}H NMR (400 MHz, $CDCl_3$) δ 8.37 (bs, 1H), 7.44–7.29 (m, 5H), 6.45 (ddd, *J* = 9.0, 5.0, 0.5 Hz, 1H), 6.22 (d, *J* = 6.0 Hz, 1H), 6.17 (ddd, *J* = 9.0, 0.5 Hz, 1H), 3.89 (s, 3H), 3.67 (dd, *J* = 6.0, 0.5 Hz, 1H); ^{13}C NMR (100 MHz, $CDCl_3$) δ 169.0, 165.3, 138.9, 138.5, 128.5, 128.1, 127.1, 126.7, 117.0, 115.8, 52.7, 52.6; IR-thin film (ν, cm^{-1}) 3225, 3103, 3059, 2953, 1746, 1673, 1635, 1587, 1449, 1437, 1240, 1193, 1158; HRMS – ESI: M+Na, calc. 266.0793, meas. 266.0793.

Methyl 5-chloro-2-oxo-2,3-dihydro-1*H*-azepine-3-carboxylate (9h)

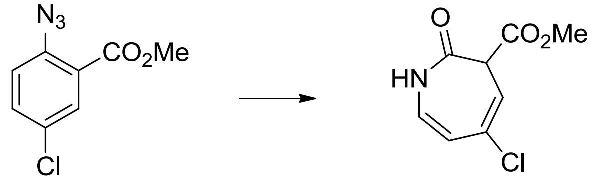

According to the general procedure, a solution of methyl 2-azido-4-chlorobenzoate (**8h**, 0.277 g, 1.36 mmol) in THF (25.8 mL) was injected at a rate of 0.267 mL/min, mixing with water at a rate of 0.200 mL/min (30 min residence time). The crude product was purified over silica gel (10–40% EtOAc/hexanes) to afford 3-methoxycarbonyl-4-chloro-3*H*-azepin-2-one (**9h**, 0.185 g, 70%) as a light yellow solid: m.p. 150–153 ºC (lit 153 ºC); ^{1}H NMR (400 MHz, d_6-DMSO) δ 10.42 (bs, 1H), 6.39 (d, *J* = 9.0 Hz, 1H), 5.86 (d, *J* = 6.5 Hz, 1H), 5.81 (d, *J* = 9.0 Hz, 1H), 3.71 (s, 3H), 3.70 (d, *J* = 6.5 Hz, 1H); ^{13}C NMR (100 MHz,

d_6-DMSO) δ 167.8, 163.3, 130.2, 129.0, 116.3, 112.0, 52.5, 51.6; IR-thin film (ν, cm^{-1}) 3228, 3126, 2950, 2844, 1751, 1674, 1627, 1599, 1478, 1434, 1369, 1330, 1264, 1238, 1162, 1030; HRMS – ESI: M+Na, calc. 224.0090, meas. 224.0077.

2,3-Dimethoxy-2,3-dihydro-1*H*-benzo[e][1,4]diazepine (9i)

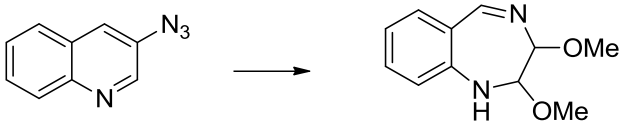

Using the flow photoreactor setup described above, but with only a single pump and no mixing, a solution of 3-azidoquinoline (**8i**, 223 mg, 1.31 mmol) in NaOMe–MeOH (1.0 M, 25.8 mL) was injected at a flow rate of 0.467 mL/min (residence time 30 min). The reactor output was concentrated under reduced pressure, taken up in CH_2Cl_2 and water and the phases were separated. The organic phase was washed with brine, dried (Na_2SO_4) and concentrated under reduced pressure to give 2,3-Dimethoxy-2,3-dihydro-1*H*-benzo[e][1,4]diazepine (**9i**, 160 mg, 59%, 75 % purity, 44% by ^{1}H-NMR): ^{1}H NMR (400 MHz, $CDCl_3$) inter alia 8.61 (d, *J* = 2.5 Hz, 1H), 7.34 (d, *J* = 8.0 Hz, 1H), 7.26 (dd, *J* = 15.5, 7.0 Hz, 1H), 6.84 (dd, *J* = 15.5, 7.0 Hz, 1H), 6.75 (d, *J* = 8.0, 1H), 5.71 (br s, 1H), 4.91 (d, *J* = 7.0, 1H), 4.51 (d, *J* = 3.5, 1H), 3.59 (s, 3H), 3.32 (s, 3H); HRMS – ESI: M+H, calc. 207.1134, meas. 207.1121; in agreement with published data. [13]

(1*S*,5*R*)-Methyl 3-oxo-2-azabicyclo[3.2.0]hept-6-ene-6-carboxylate (10)

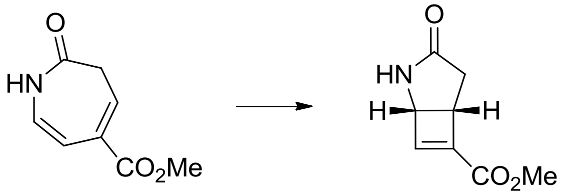

A solution of **9a** (90 mg, 0.538 mmol) in THF (10 mL) and water (7.5 mL) was injected into the flow photoreactor described above. After irradiation for 4.5 h, the contents of the reactor were pumped out and extracted with EtOAc. The combined organic extracts were washed with brine, dried (Na_2SO_4) and

concentrated under reduced pressure to afford a mixture of **9a** and (1*S*,5*R*)-methyl 3-oxo-2-azabicyclo[3.2.0]hept-6-ene-6-carboxylate (**10**, 81 mg, 90%, 50% conversion by ^{1}H-NMR): ^{1}H NMR (400 MHz, d_6-acetone) 7.36 (bs, 1H), 6.96 (s, 1H), 4.41 (d, J = 4.0, 1H), 3.73 (s, 3H), 3.73–3.70 (m, 1H), 2.45 (dd, J = 18.0, 11.0, 1H); 2.22 (dd, J = 18.0, 3.0, 1H); ^{13}C NMR (100 MHz, d_6-acetone) 177.5, 162.3, 149.8, 144.4, 54.5, 51.8, 41.2, 33.3.

4. ^{1}H and ^{13}C NMR data

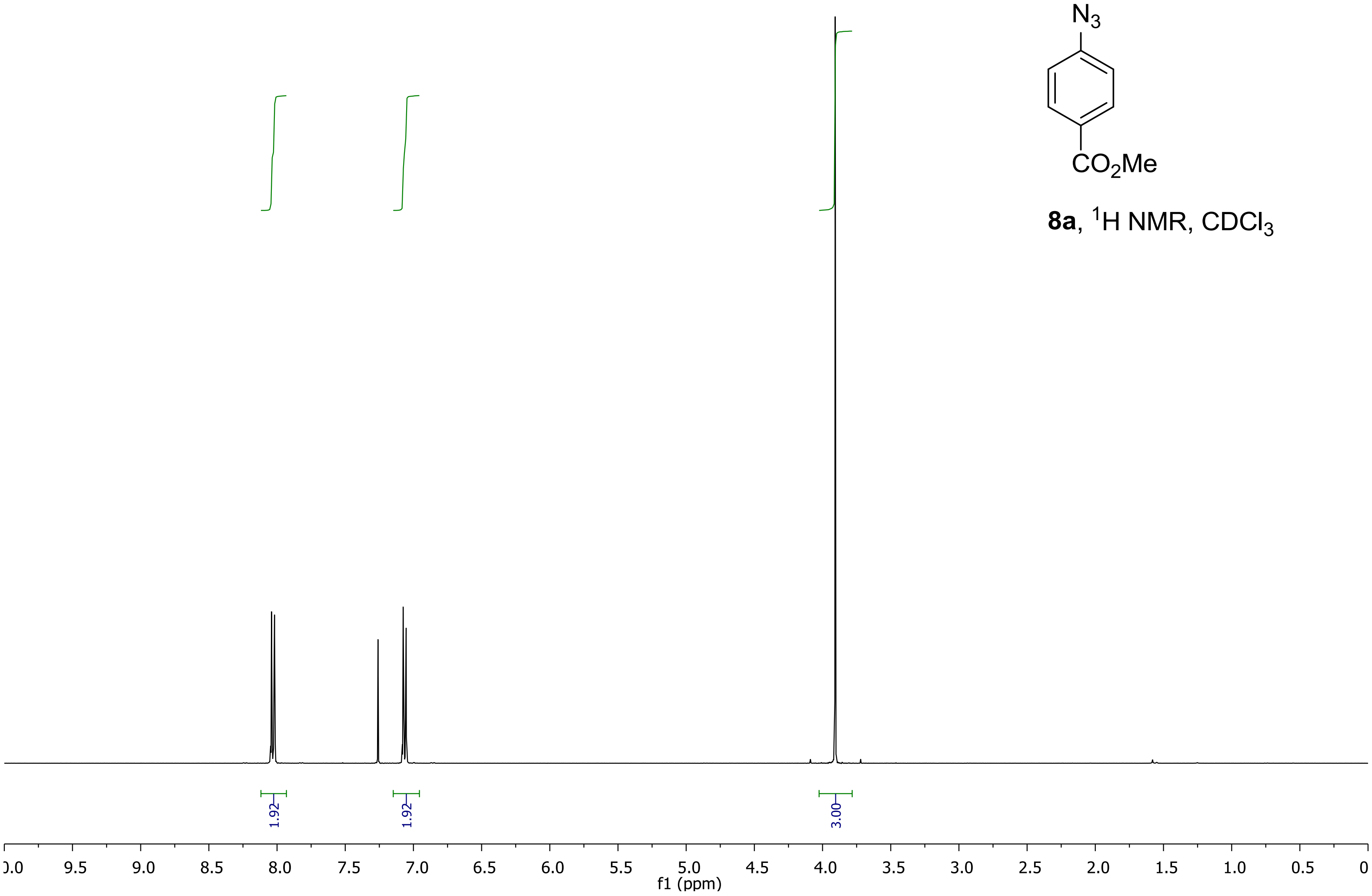

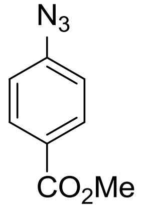

8a, ^{13}C NMR, $CDCl_3$

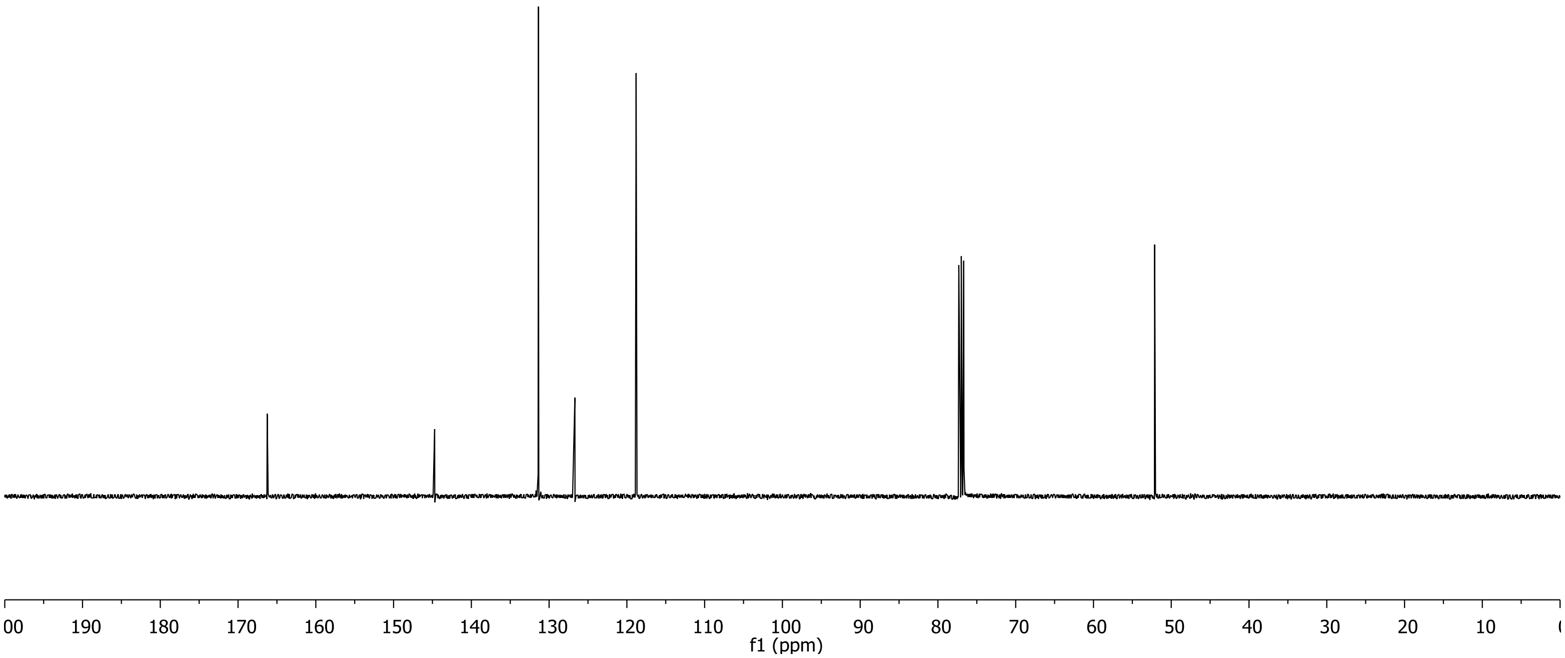

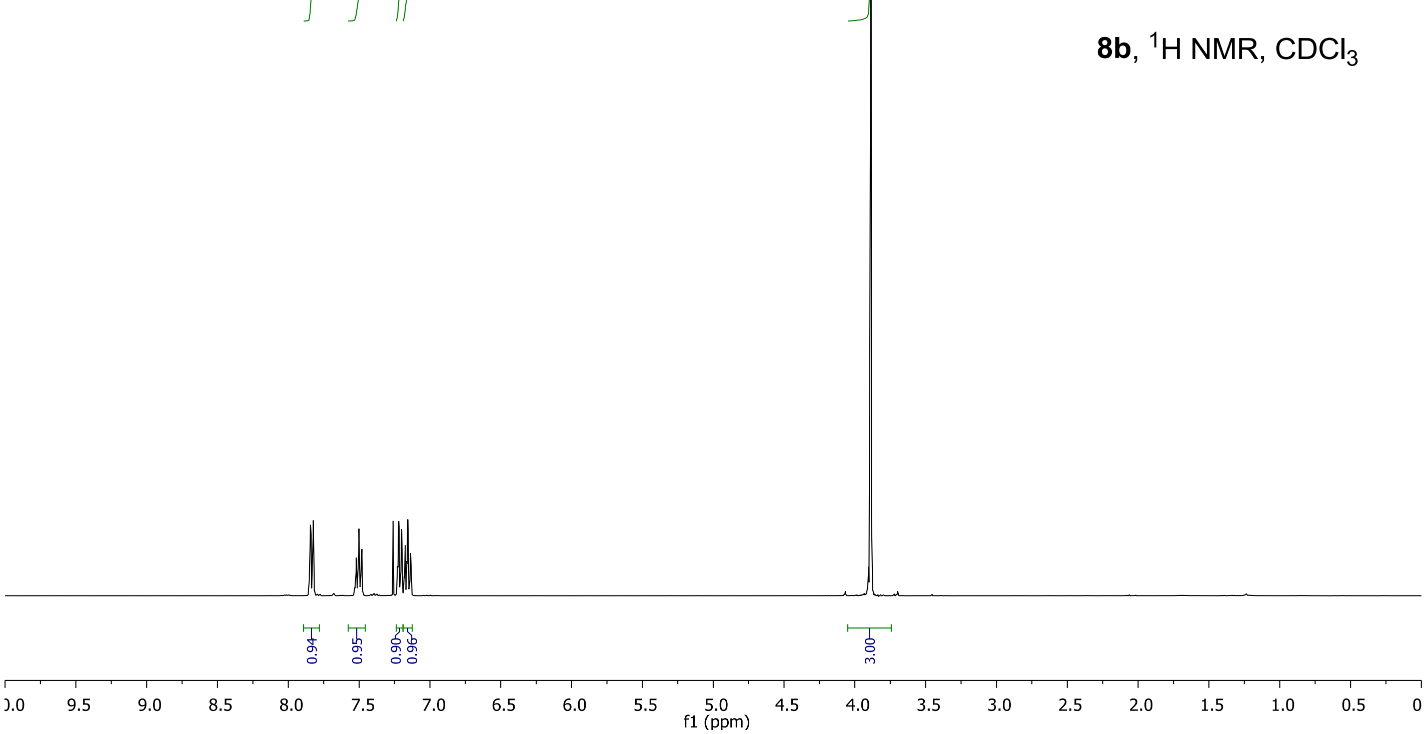

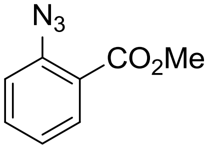

8b, ^{1}H NMR, $CDCl_3$

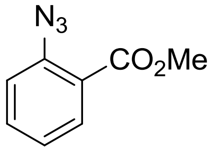

8b, ^{13}C NMR, $CDCl_3$

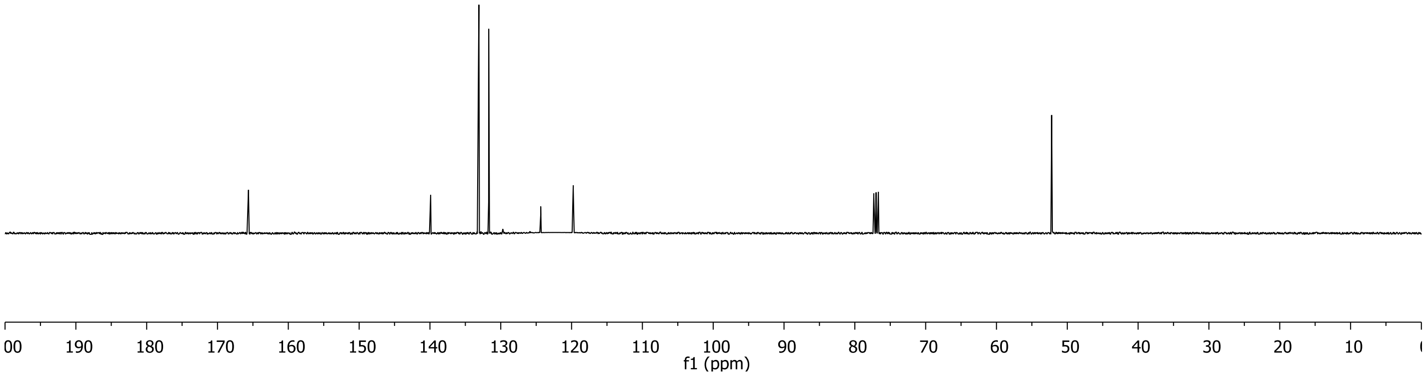

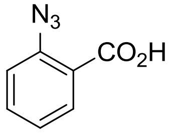

8c, ^{1}H NMR, d_6-DMSO

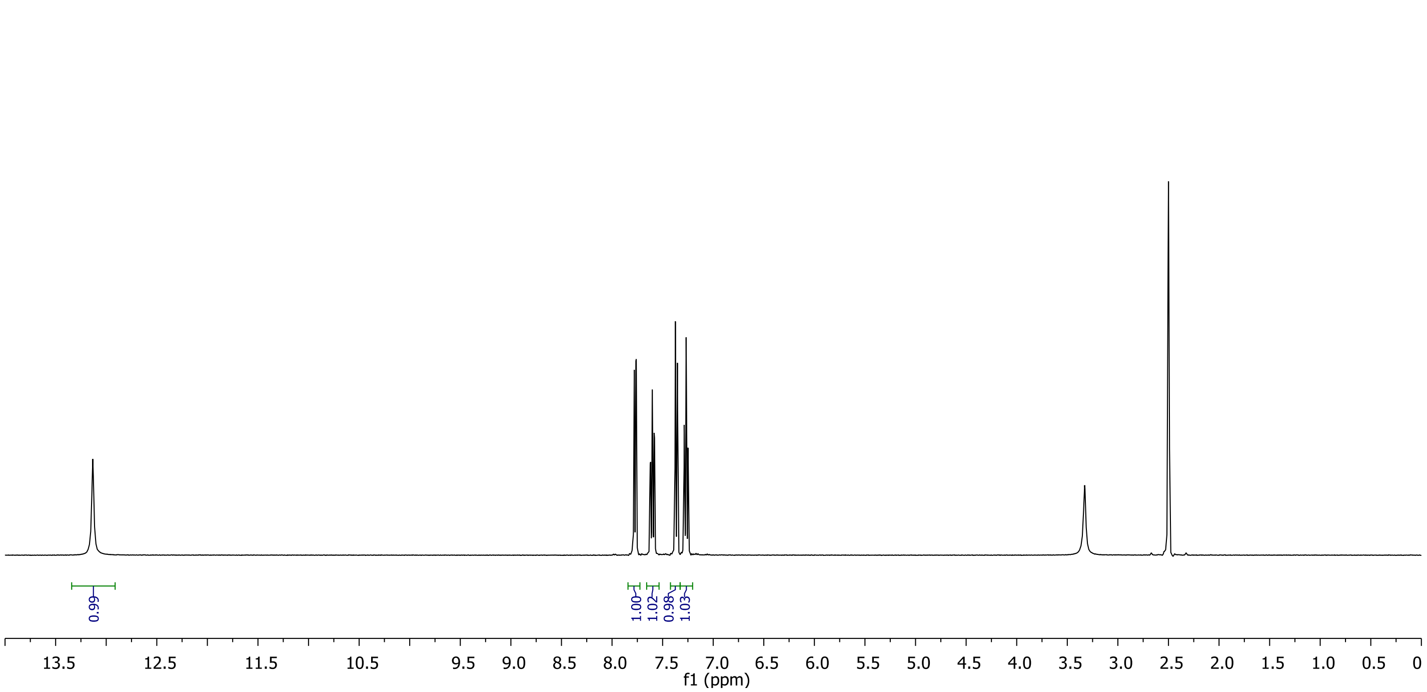

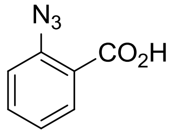

8c, ^{13}C NMR, d_6-DMSO

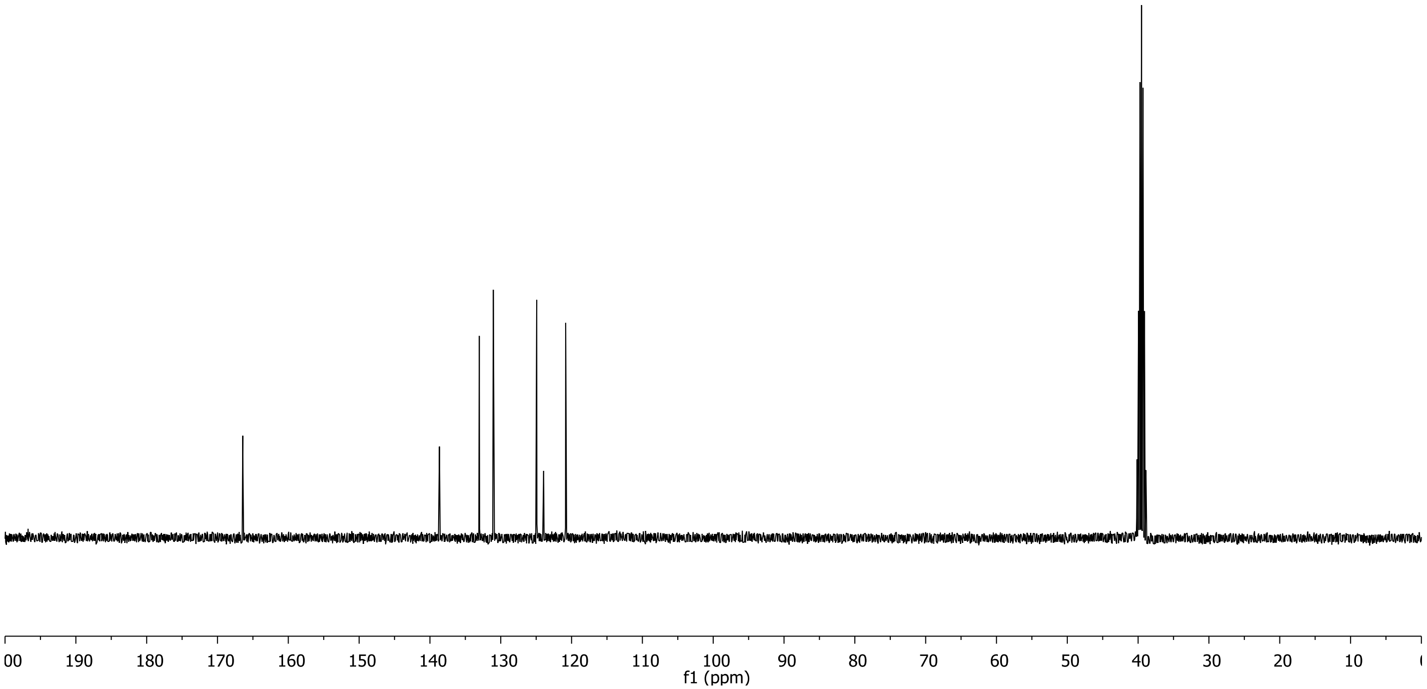

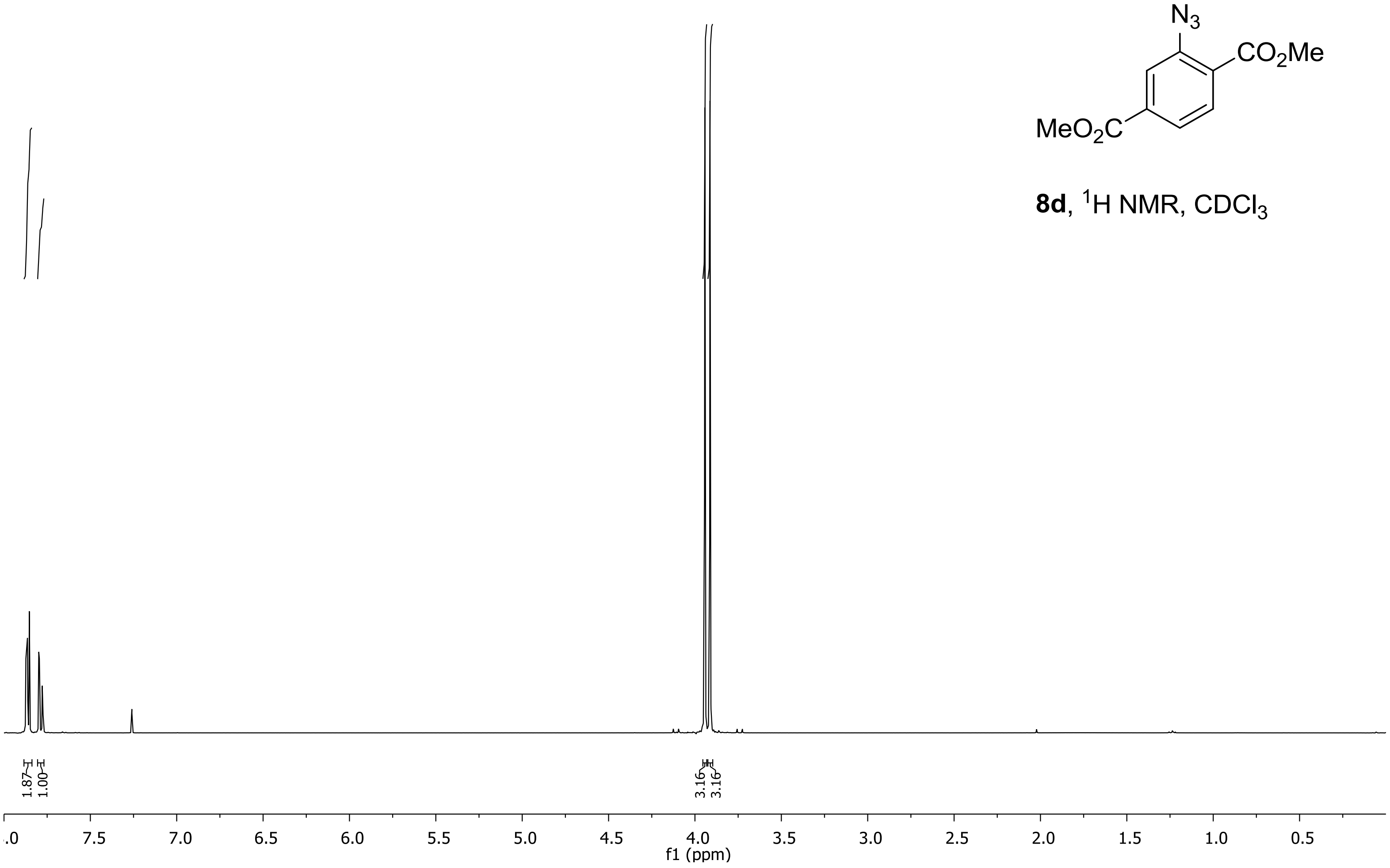
N3
CO2Me
MeO2C
8d, 1H NMR, CDCl3
1.87
1.00
3.16
3.16
.0
7.5
7.0
6.5
6.0
5.5
5.0
4.5
4.0
3.5
3.0
2.5
2.0
1.5
1.0
0.5
f1 (ppm)

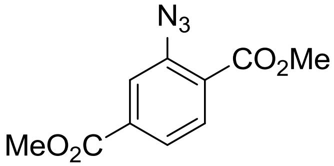

8d, ^{13}C NMR, $CDCl_3$

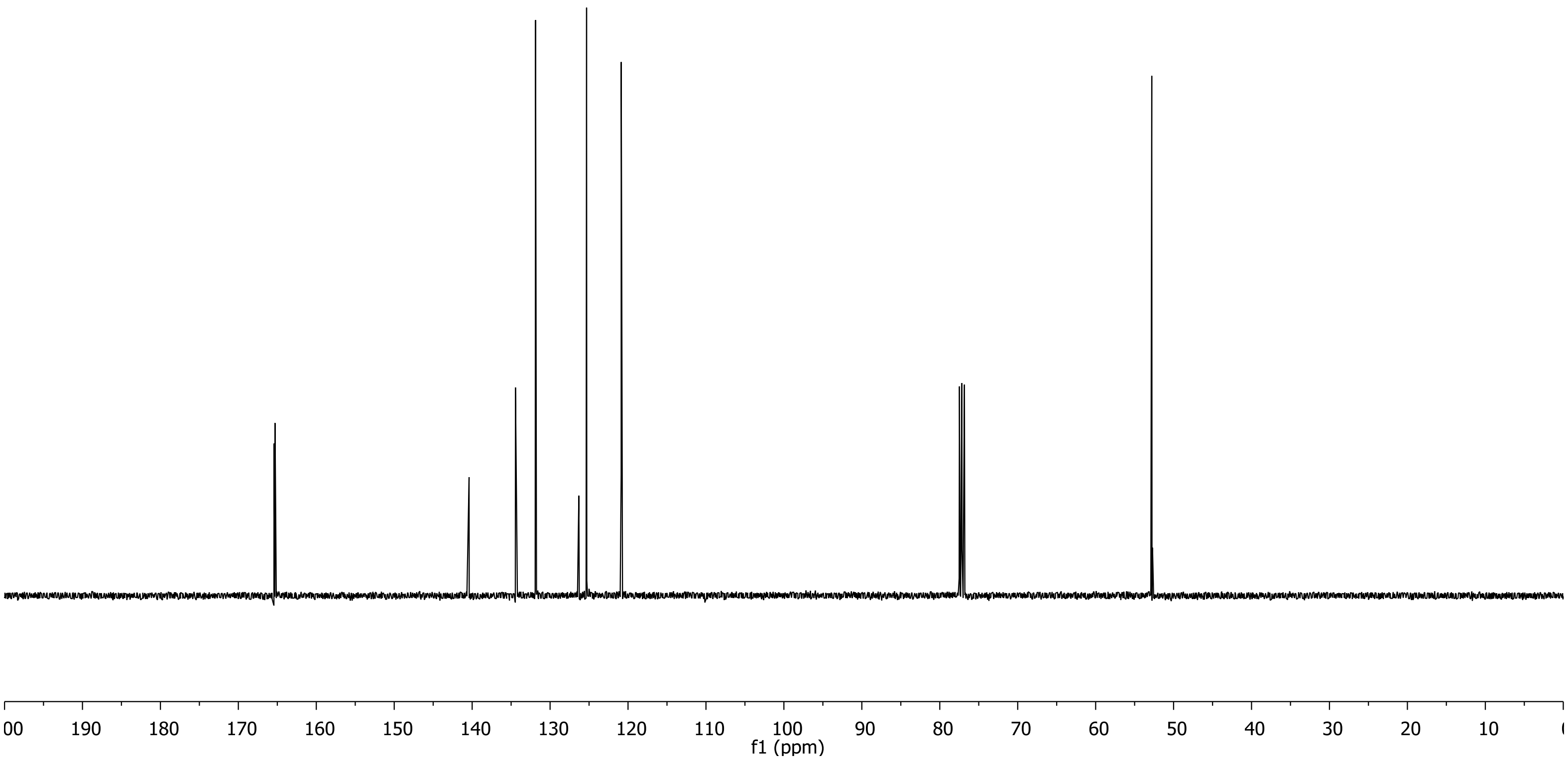

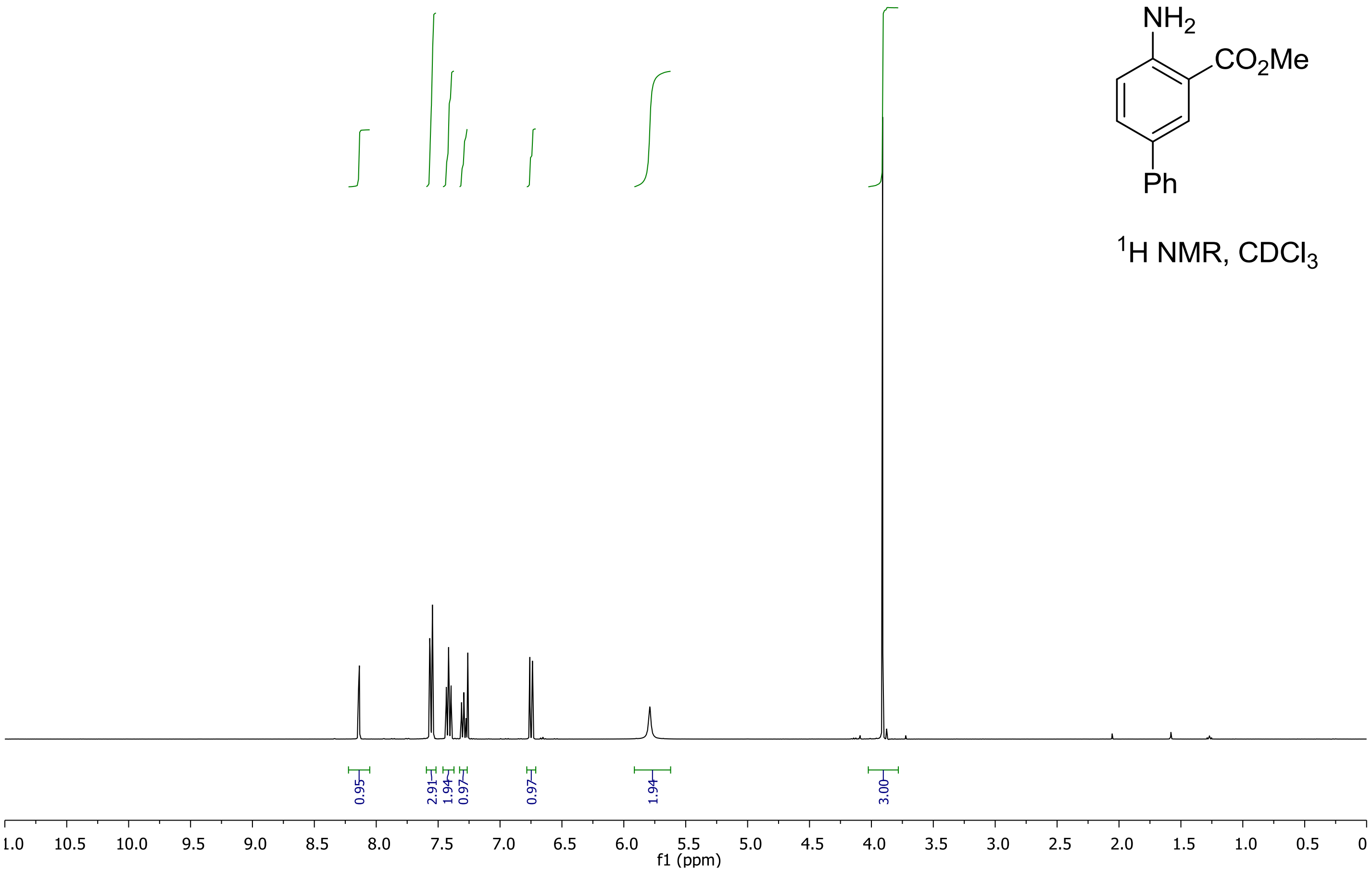
NH2
CO2Me
Ph
1H NMR, CDCl3
0.95
2.91
1.94
0.97
0.97
1.94
3.00
11.0 10.5 10.0 9.5 9.0 8.5 8.0 7.5 7.0 6.5 6.0 5.5 5.0 4.5 4.0 3.5 3.0 2.5 2.0 1.5 1.0 0.5 0
f1 (ppm)

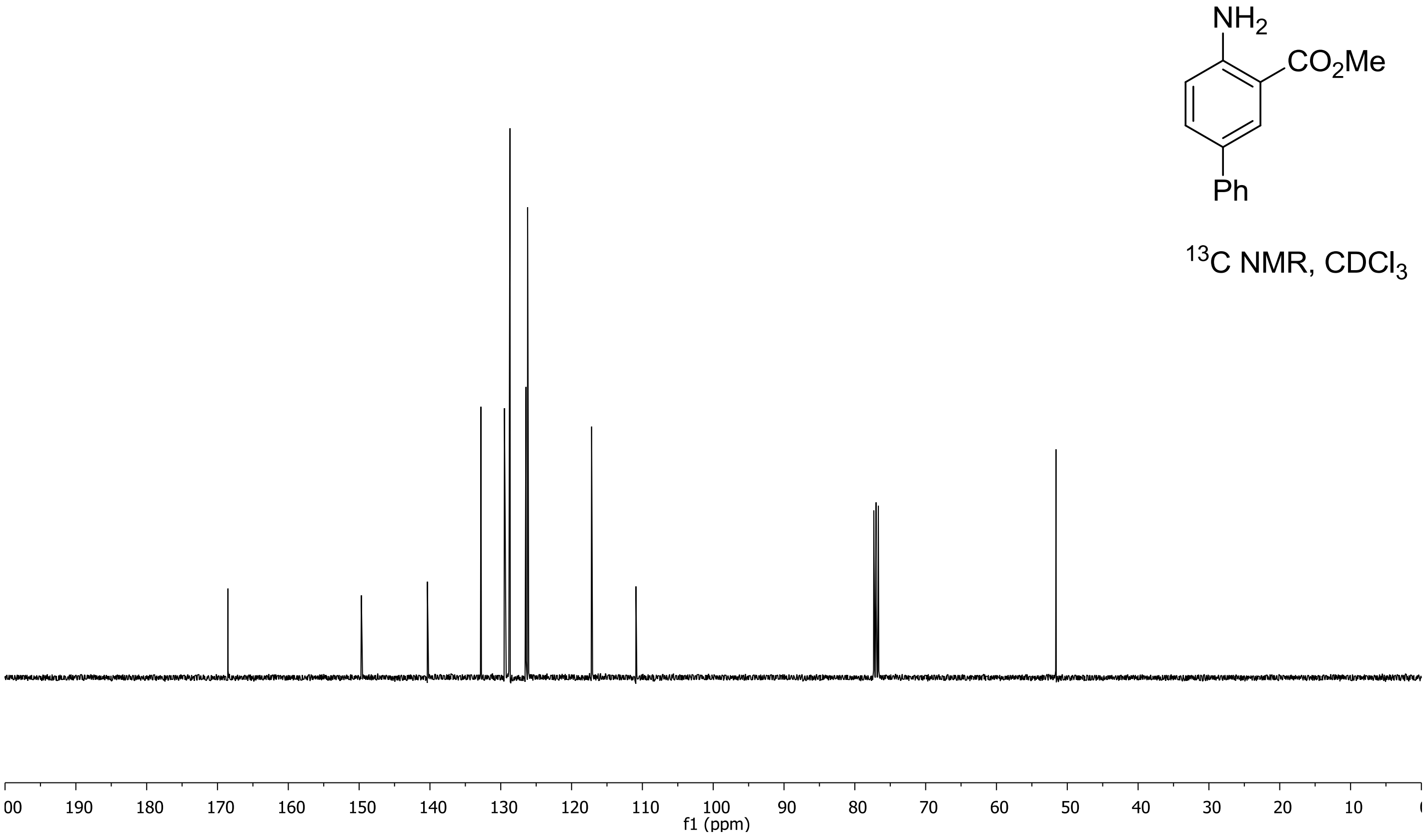
NH2
CO2Me
Ph
13C NMR, CDCl3
00
190
180
170
160
150
140
130
120
110
100
90
80
70
60
50
40
30
20
10
f1 (ppm)

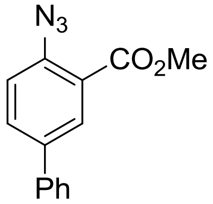

8e, ^{1}H NMR, $CDCl_3$

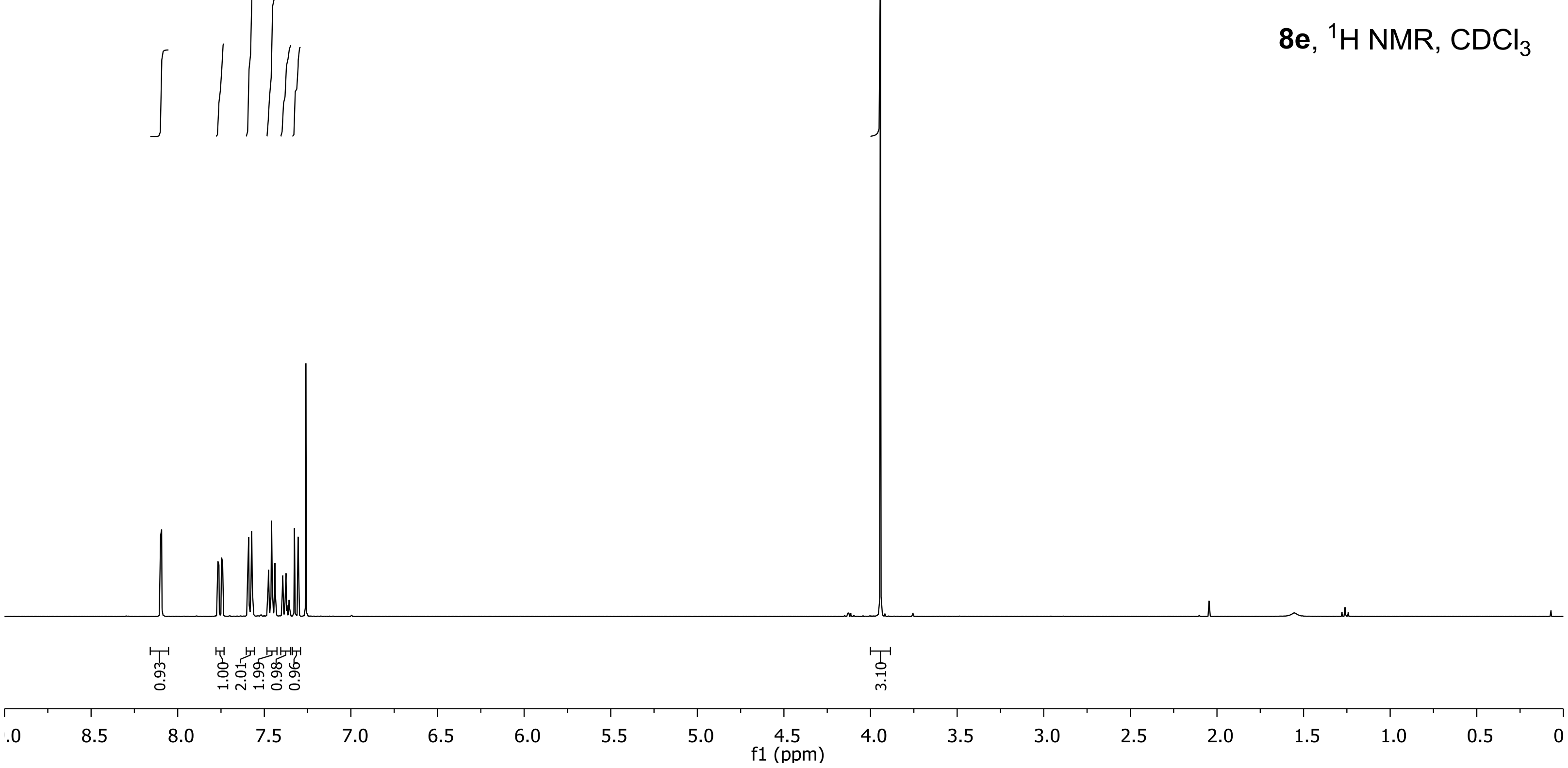

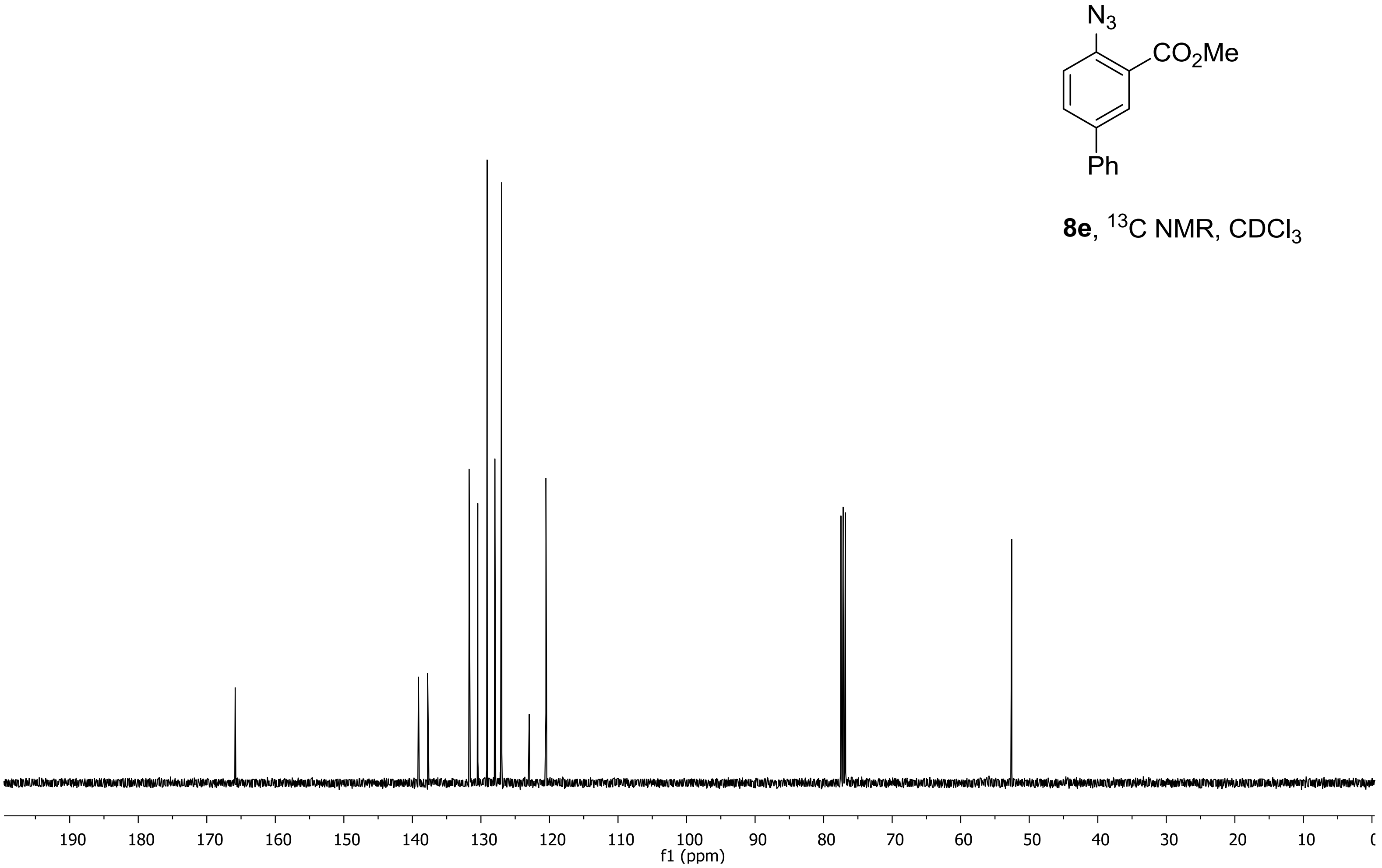
N3
CO2Me
Ph
8e, 13C NMR, CDCl3
190 180 170 160 150 140 130 120 110 100 90 80 70 60 50 40 30 20 10
f1 (ppm)

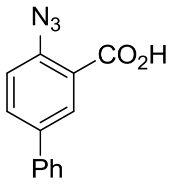

8f, ^1H NMR, $CDCl_3$

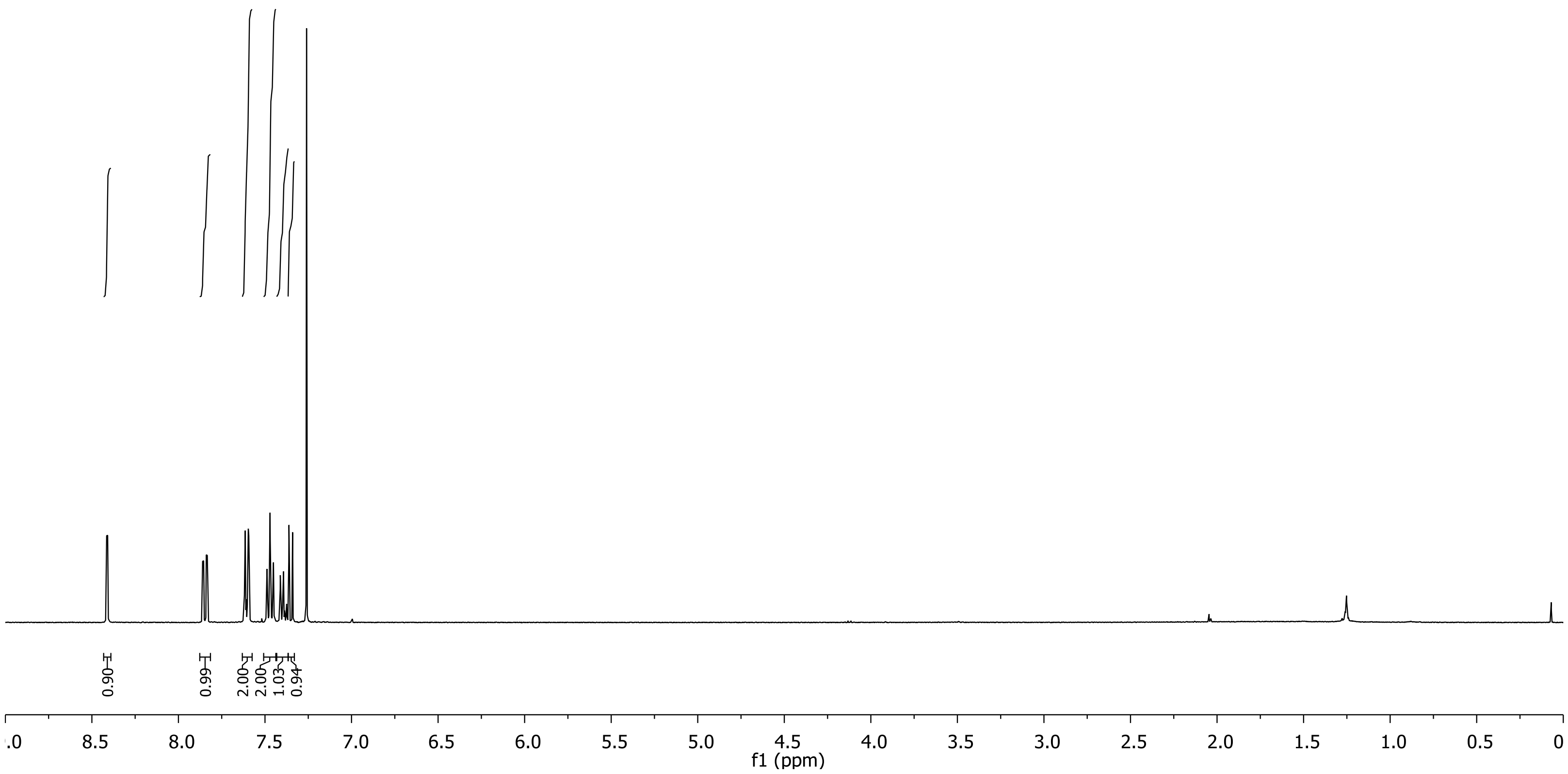

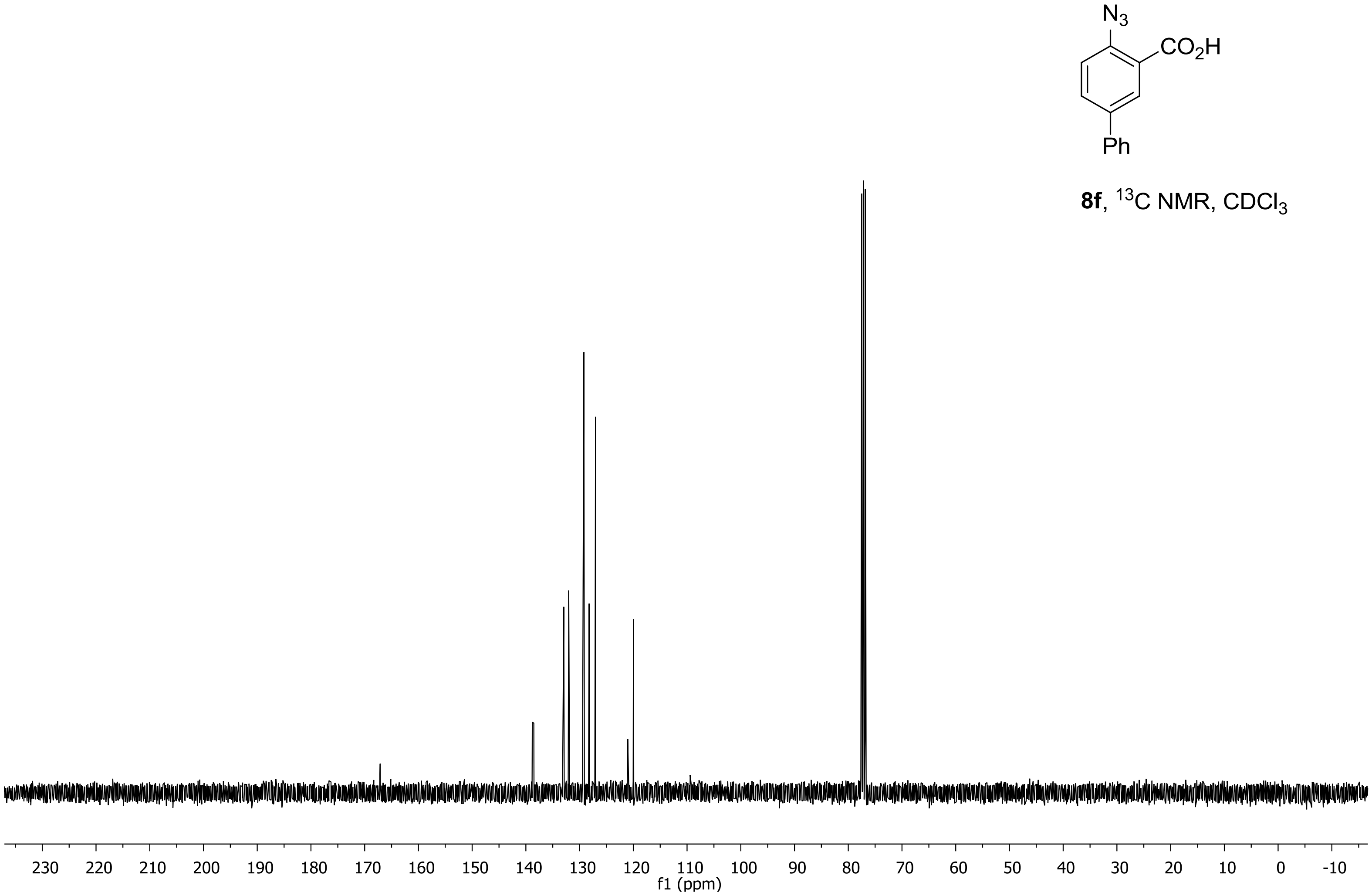

8f, ^{13}C NMR, $CDCl_3$

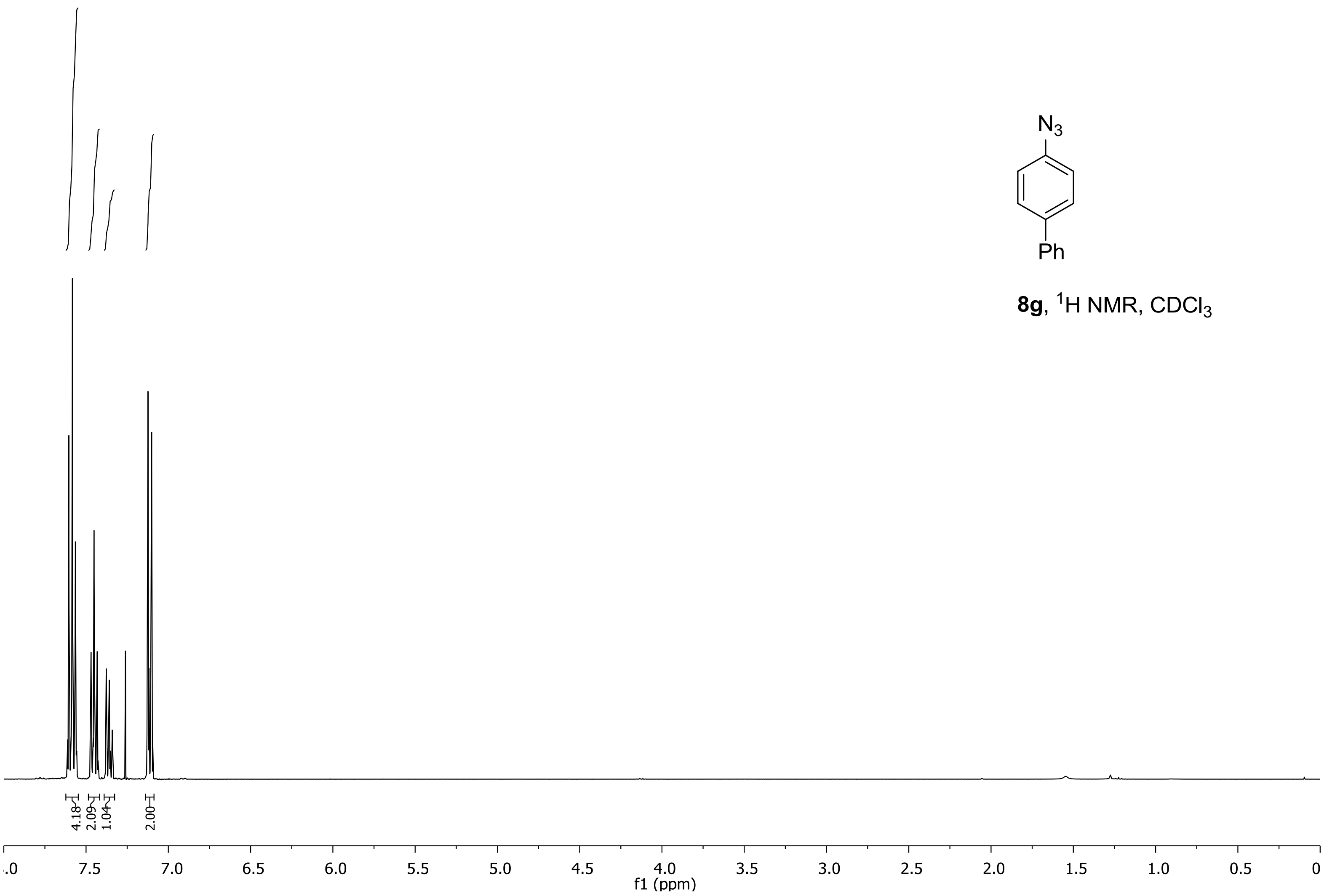

8g, ^{1}H NMR, $CDCl_3$

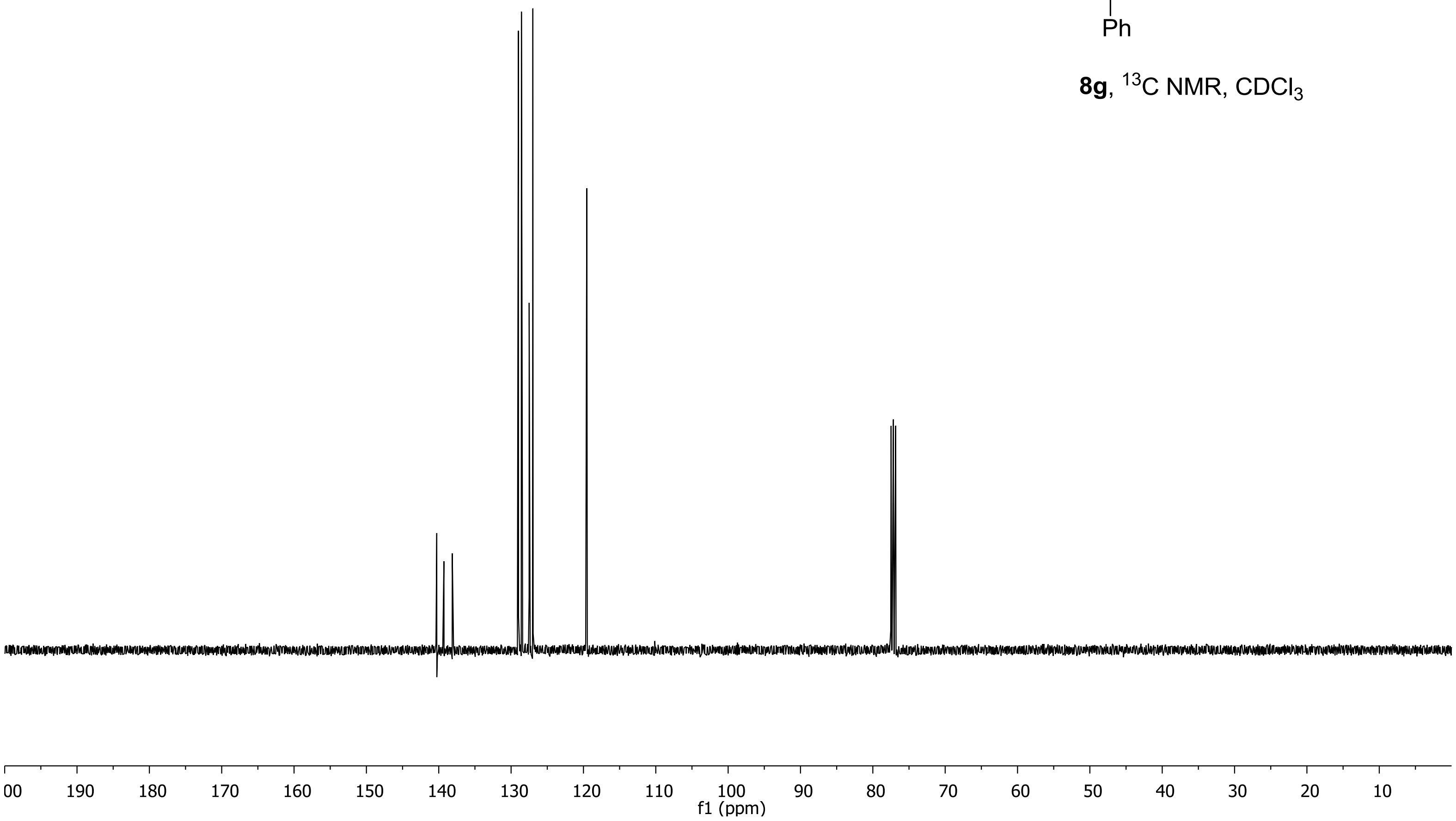

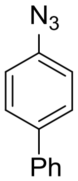

8g, ^{13}C NMR, $CDCl_3$

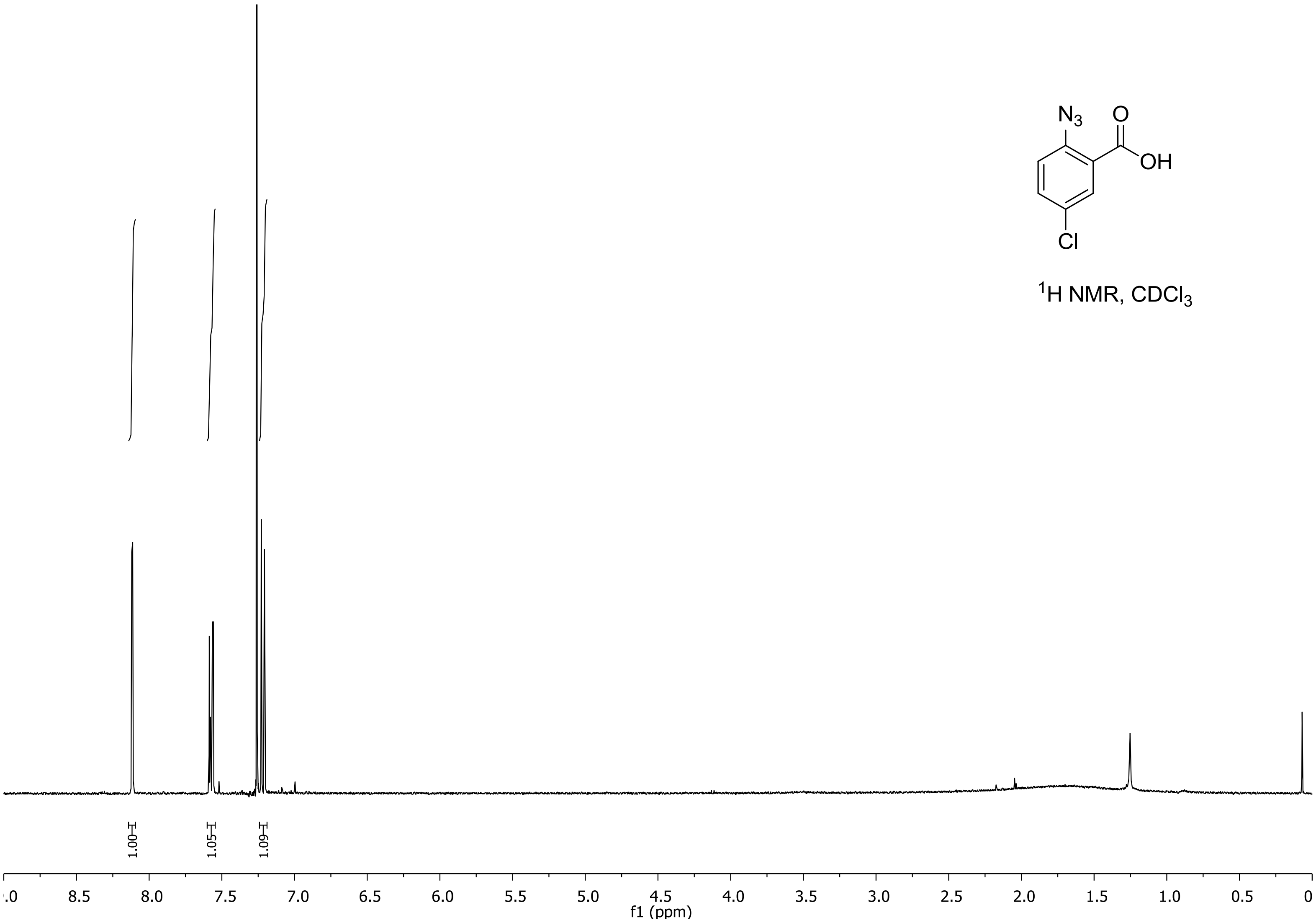

N3
O
OH
Cl
1H NMR, CDCl3
1.00
1.05
1.09
.0
8.5
8.0
7.5
7.0
6.5
6.0
5.5
5.0
4.5
4.0
3.5
3.0
2.5
2.0
1.5
1.0
0.5
0
f1 (ppm)

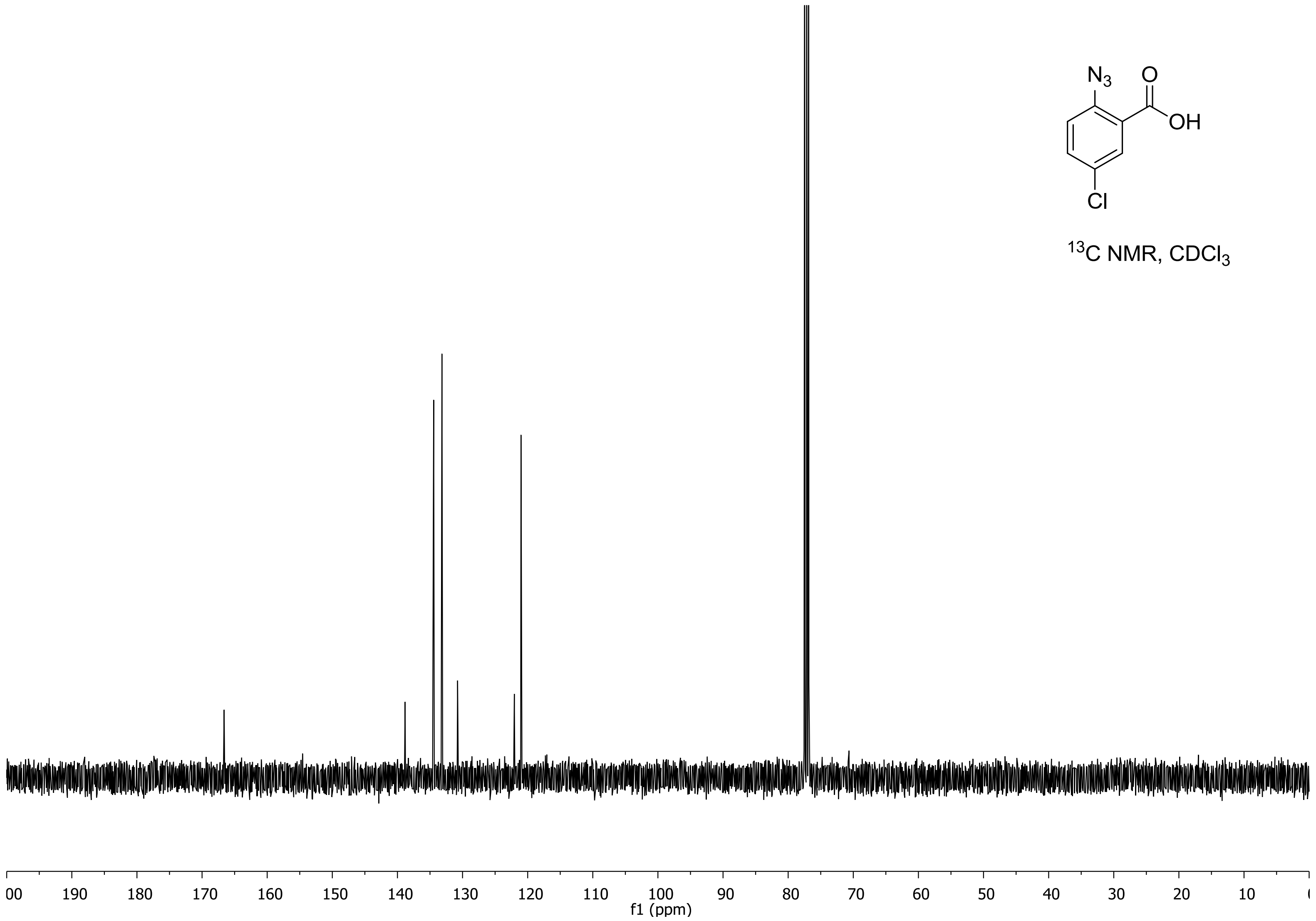
N3
O
OH
Cl
13C NMR, CDCl3
190
180
170
160
150
140
130
120
110
100
90
80
70
60
50
40
30
20
10
f1 (ppm)

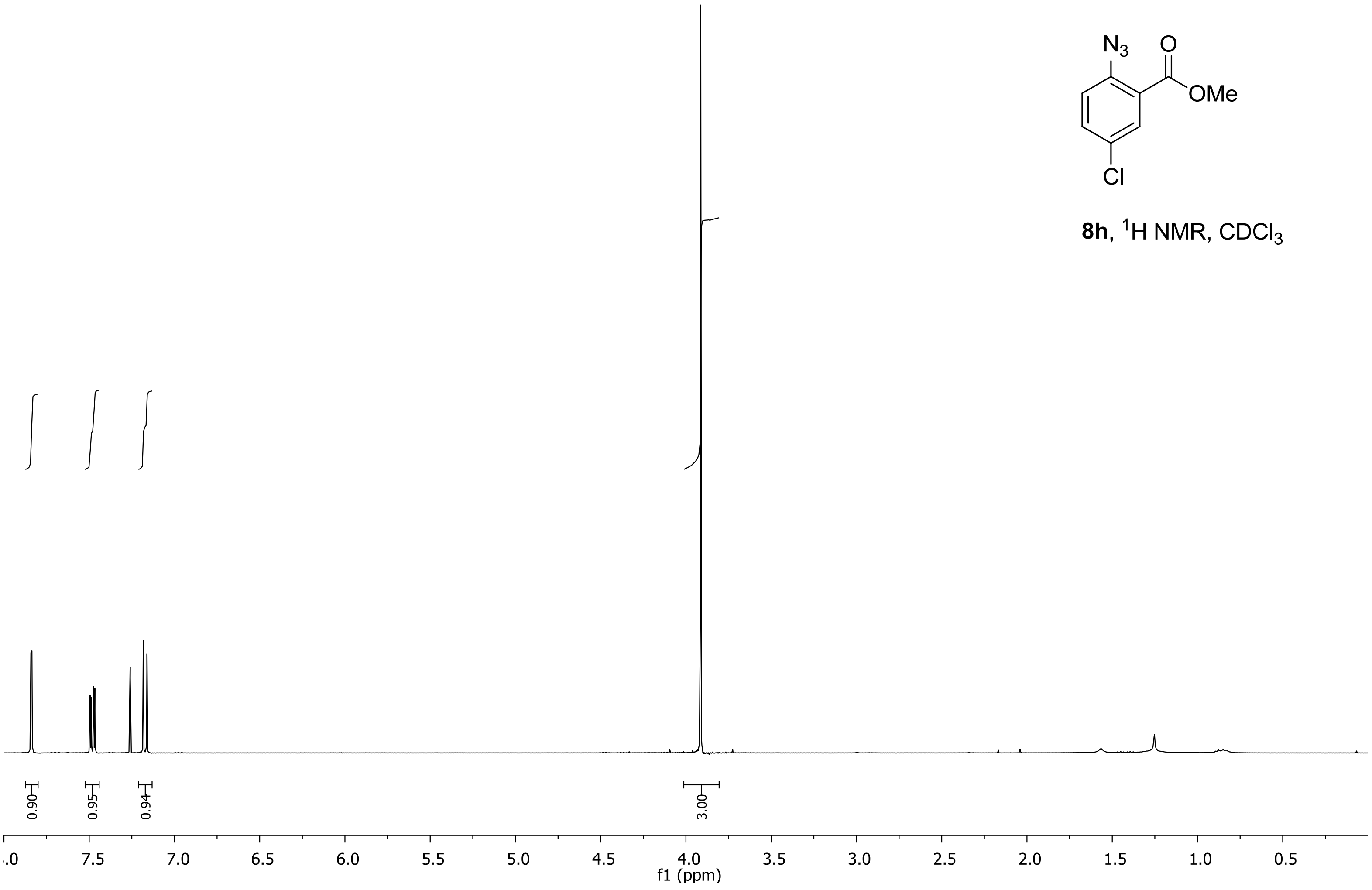
N3
O
OMe
Cl
8h, 1H NMR, CDCl3
0.90
0.95
0.94
3.00
.0
7.5
7.0
6.5
6.0
5.5
5.0
4.5
4.0
3.5
3.0
2.5
2.0
1.5
1.0
0.5
f1 (ppm)

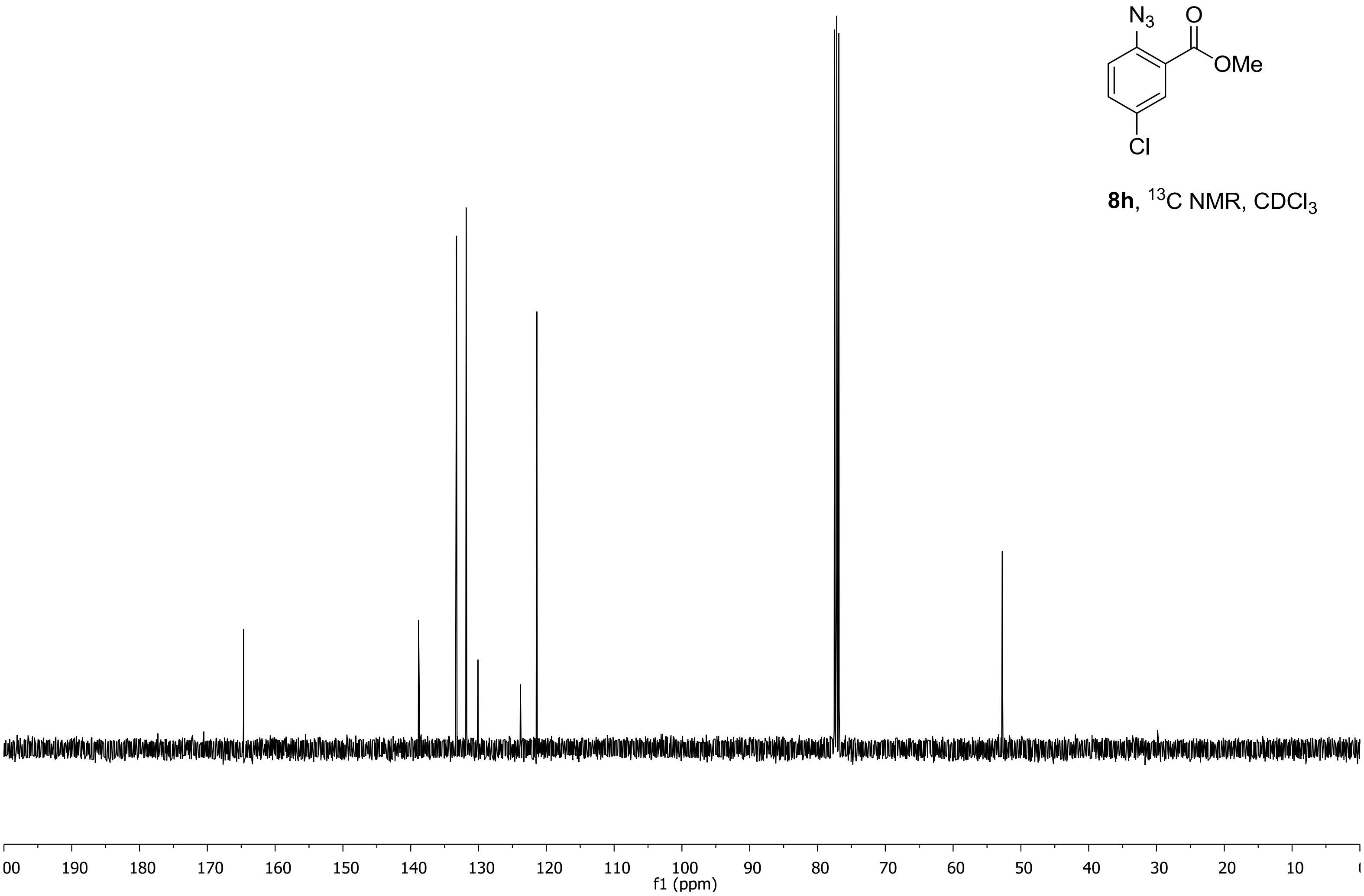

8h, ^{13}C NMR, $CDCl_3$

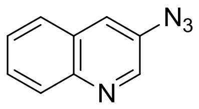

8i, ^{1}H NMR, $CDCl_3$

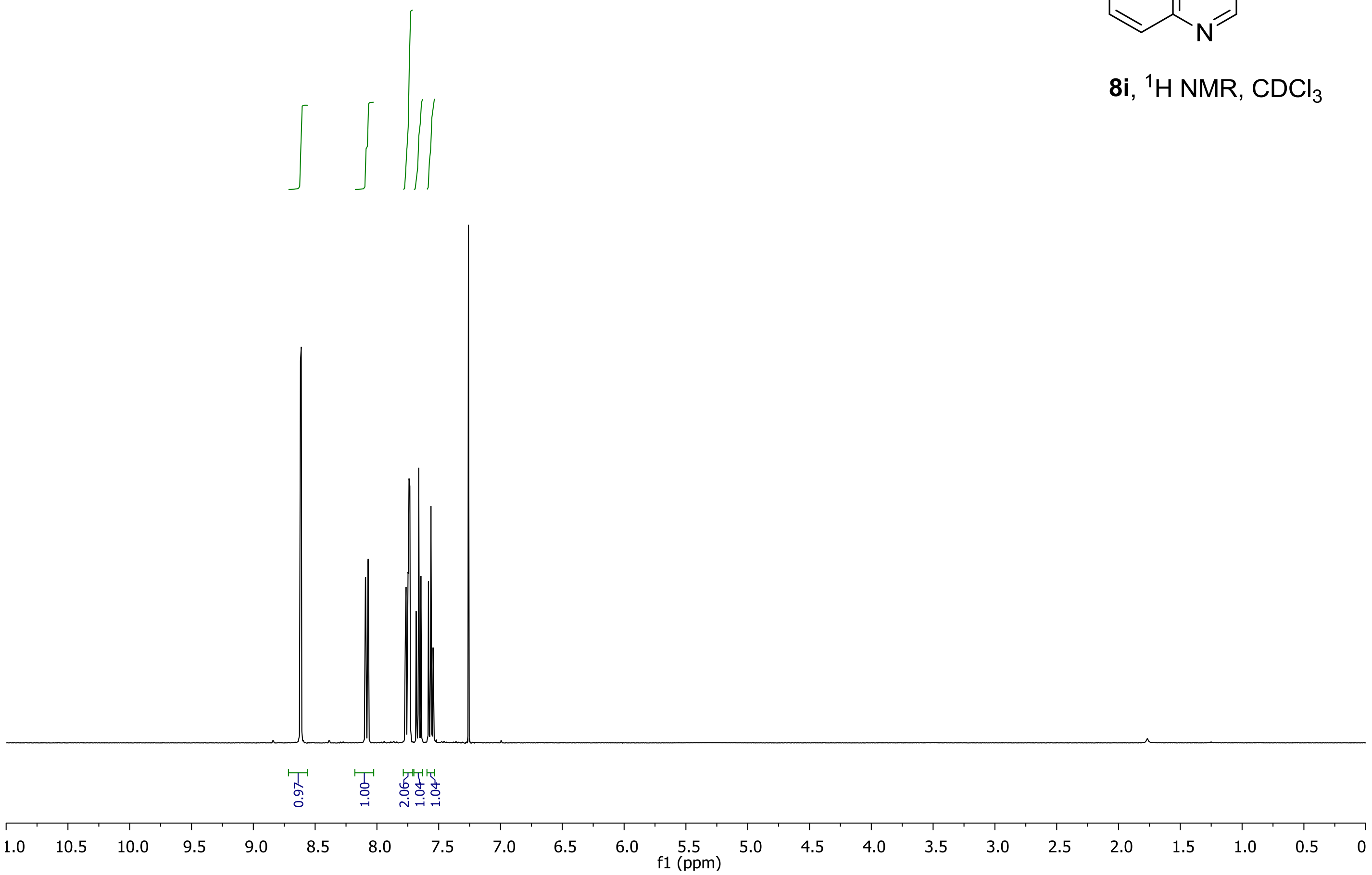

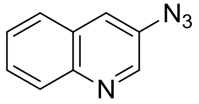

8i, ^{13}C NMR, $CDCl_3$

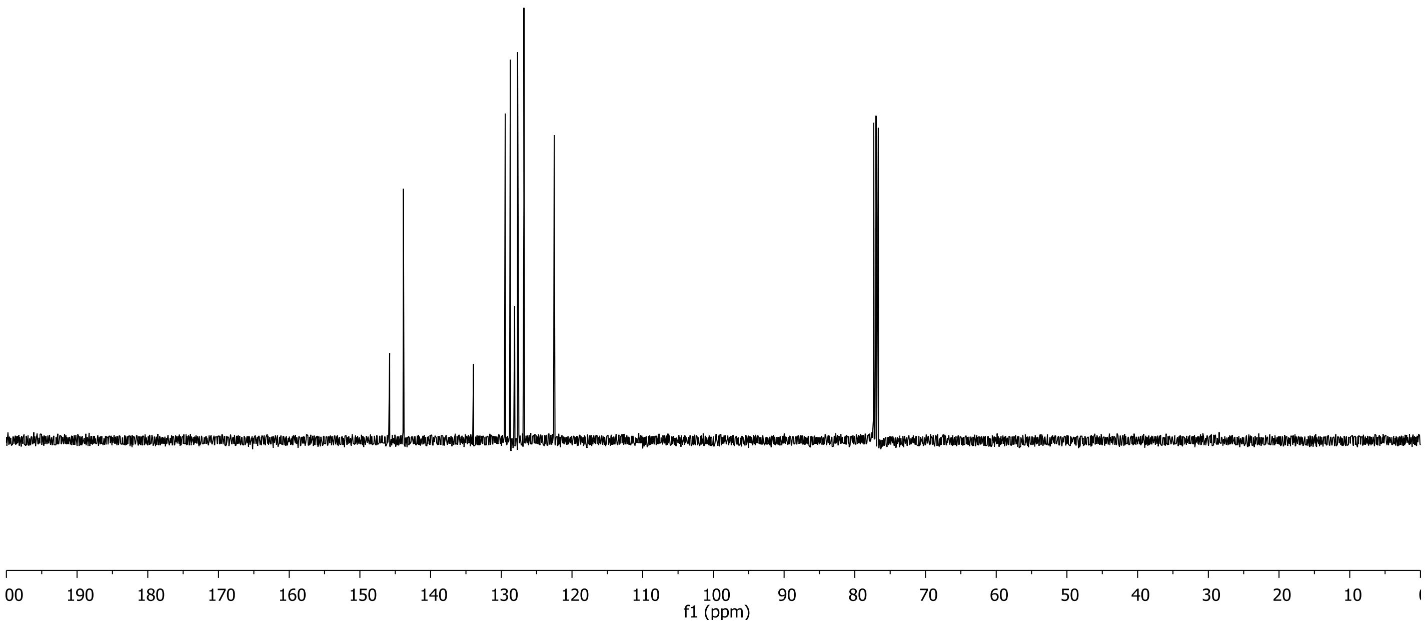

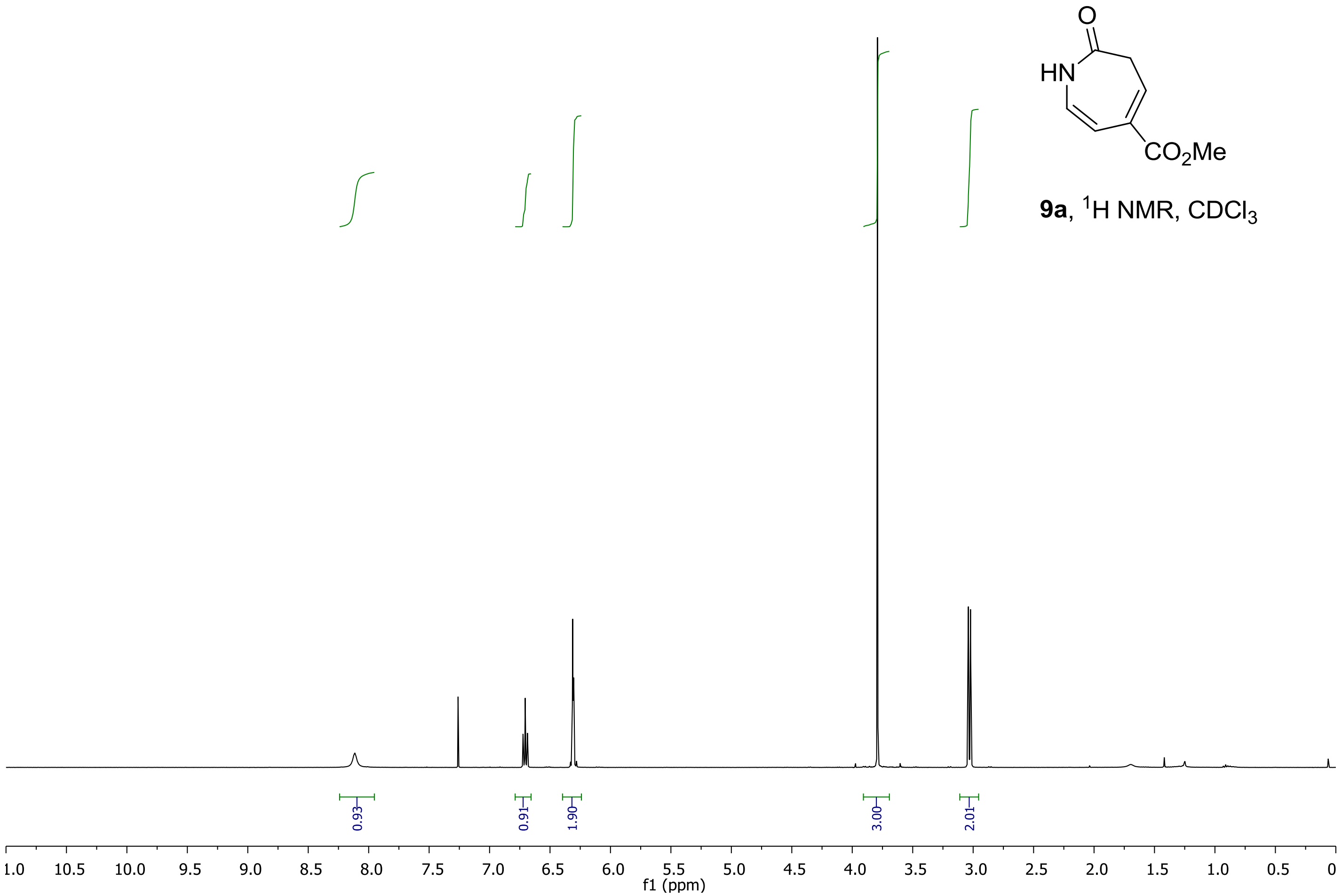

9a, ^{1}H NMR, $CDCl_3$

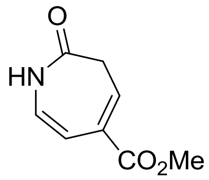

9a, ^{13}C NMR, $CDCl_3$

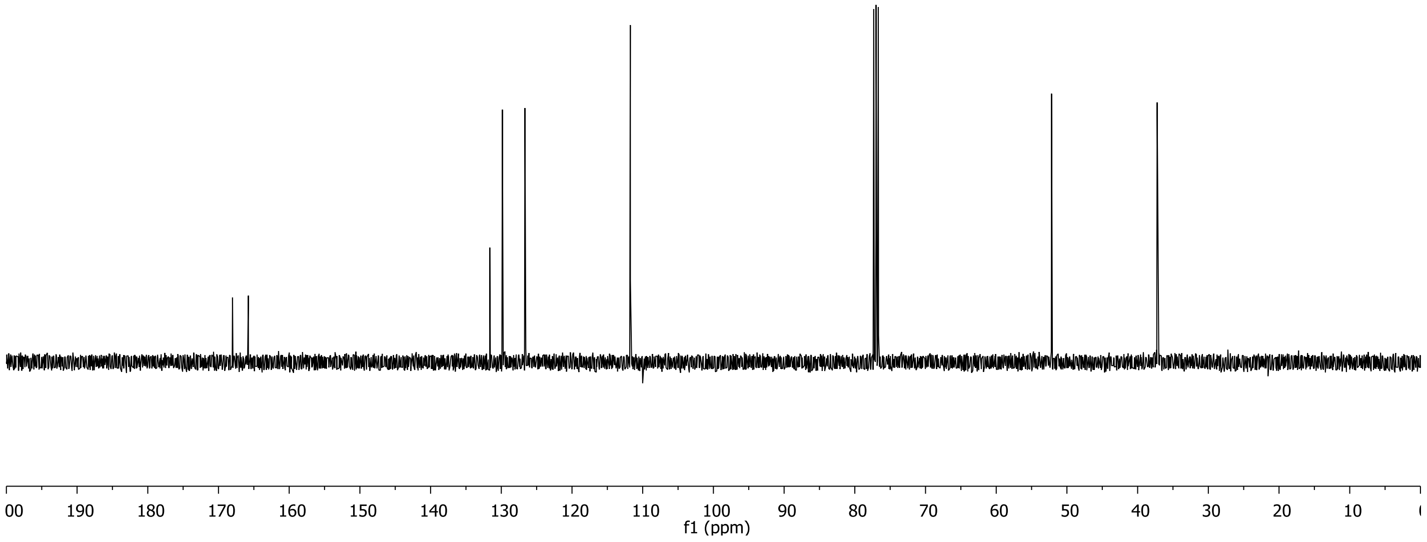

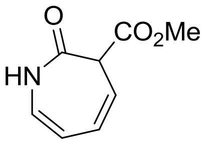

9b, ^1H NMR, $CDCl_3$

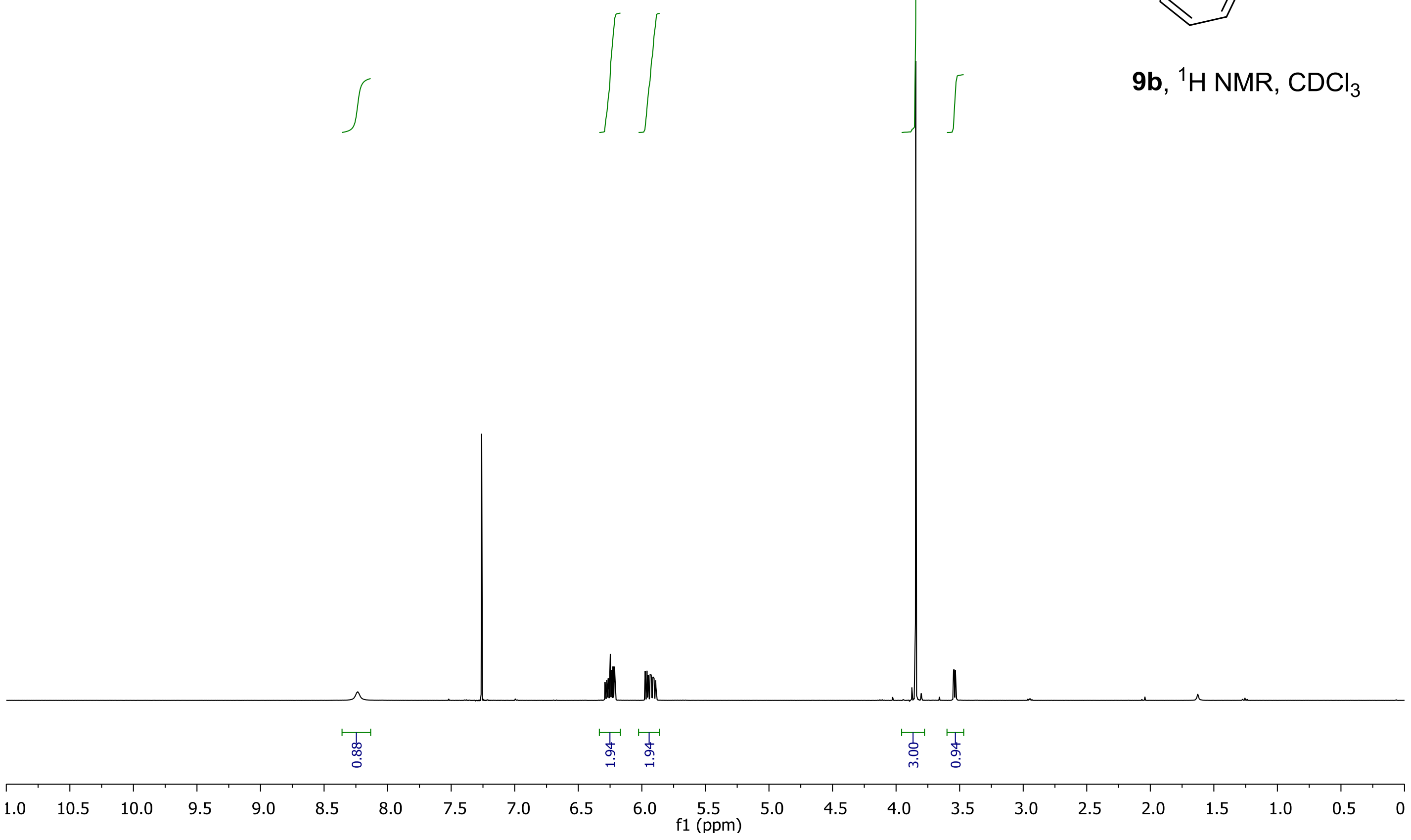

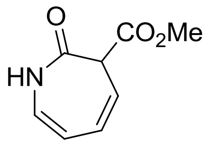

9b, ^{13}C NMR, $CDCl_3$

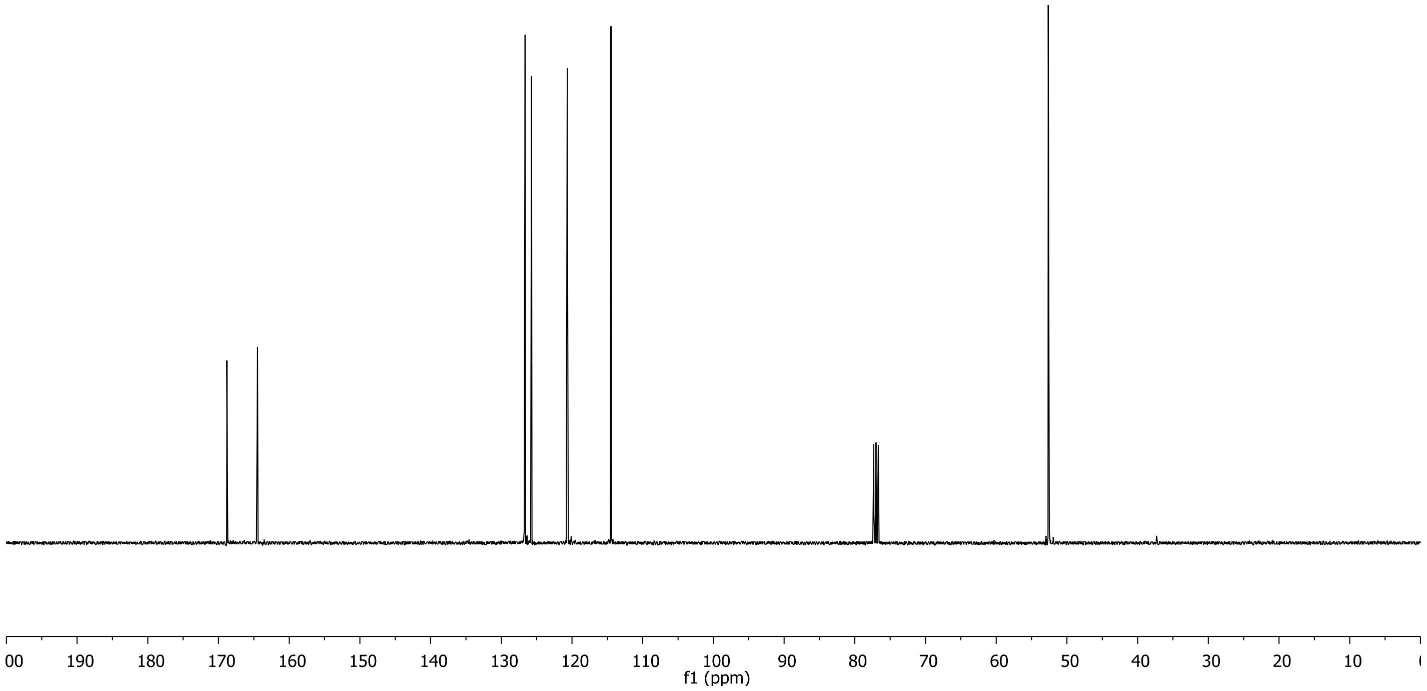

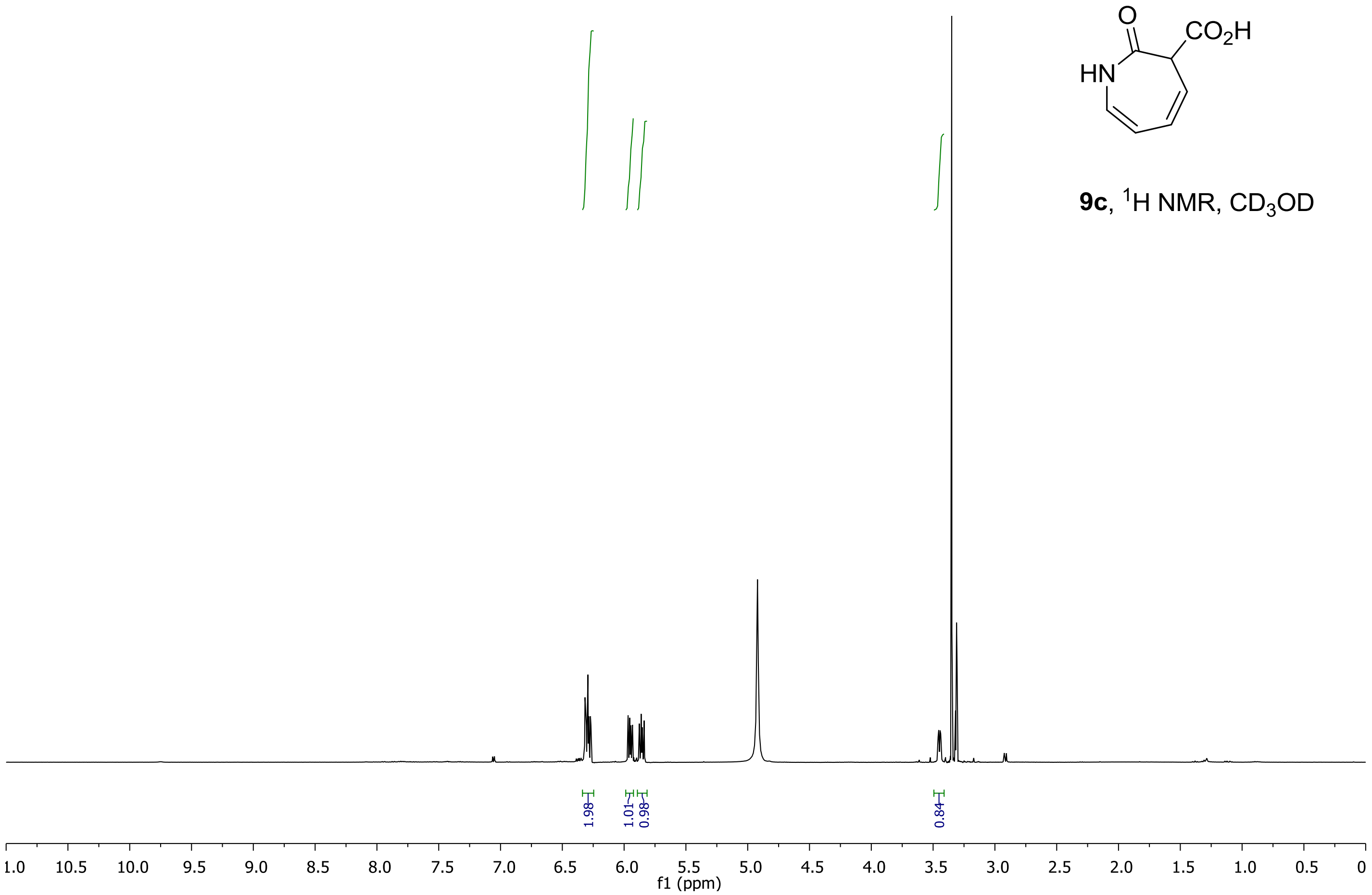

9c, ^{1}H NMR, CD_3OD

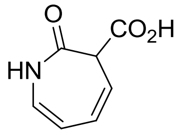

9c, ^{13}C NMR, CD_3OD

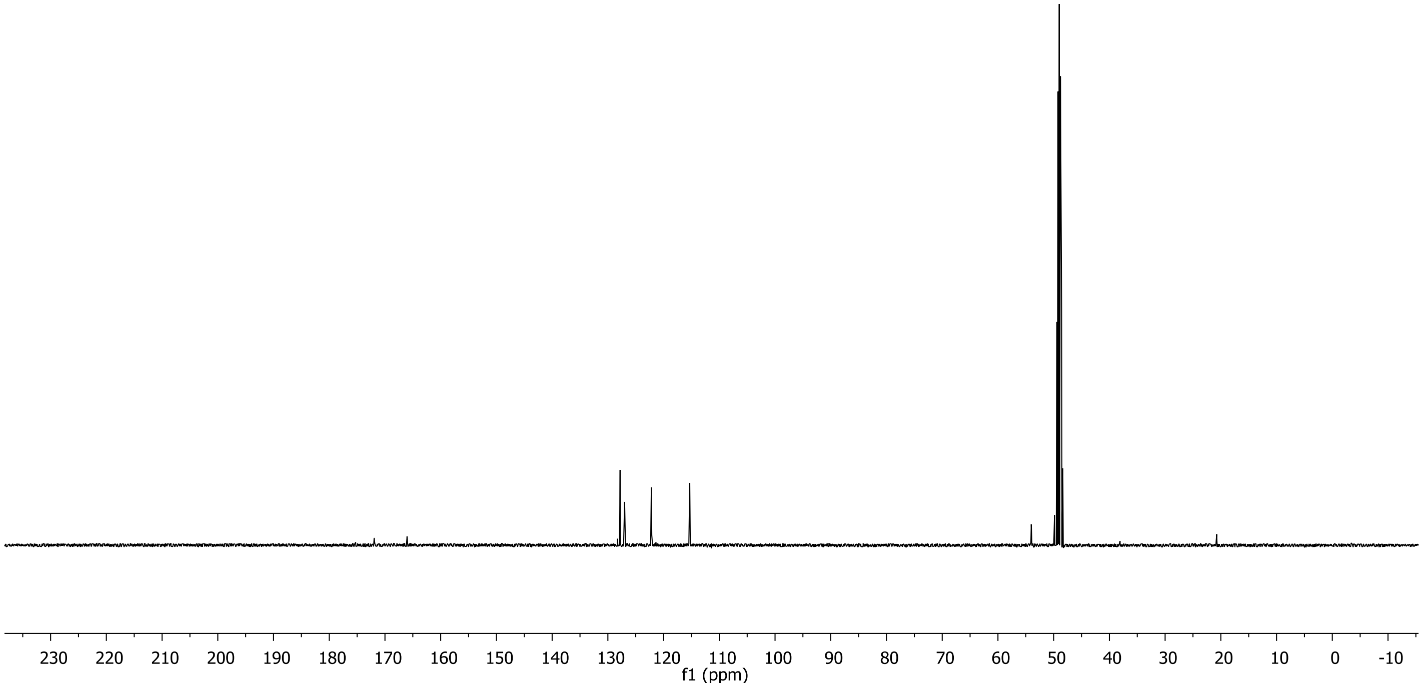

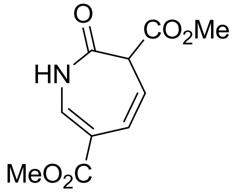

9d, ^{1}H NMR, $CDCl_3$

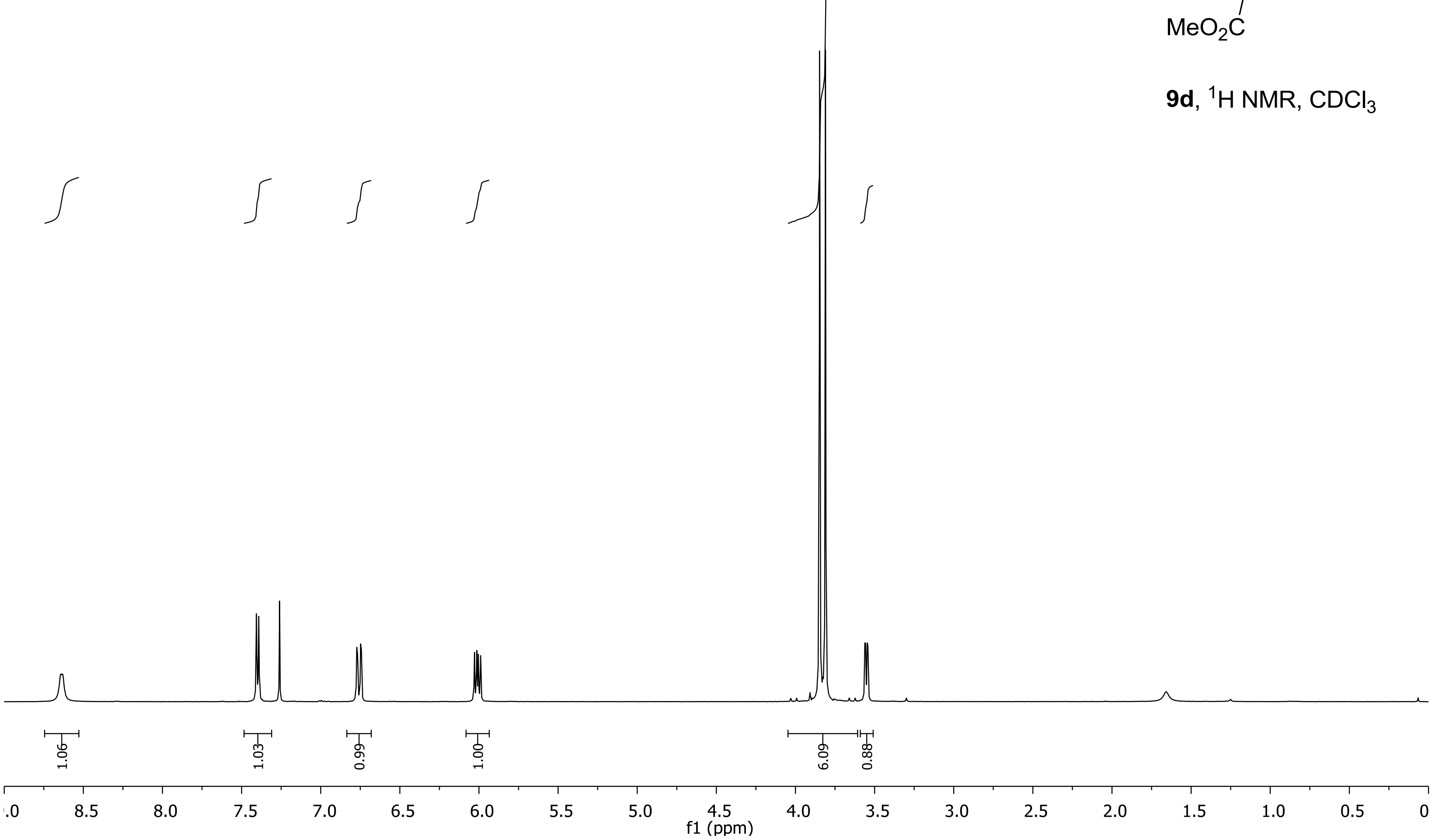

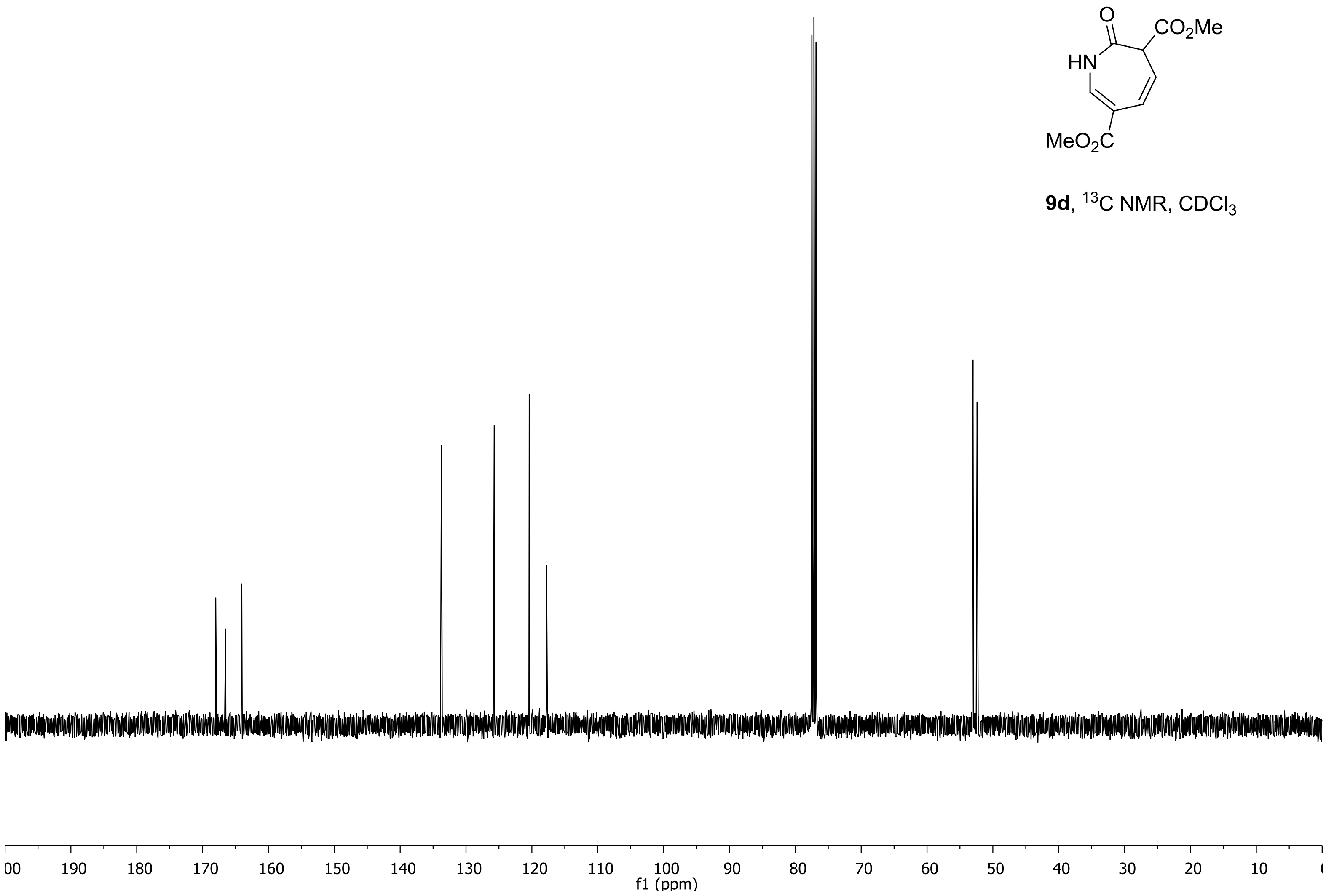
O
CO_2Me
HN
MeO_2C
9d, ^{13}C NMR, $CDCl_3$
00 190 180 170 160 150 140 130 120 110 100 90 80 70 60 50 40 30 20 10
f1 (ppm)

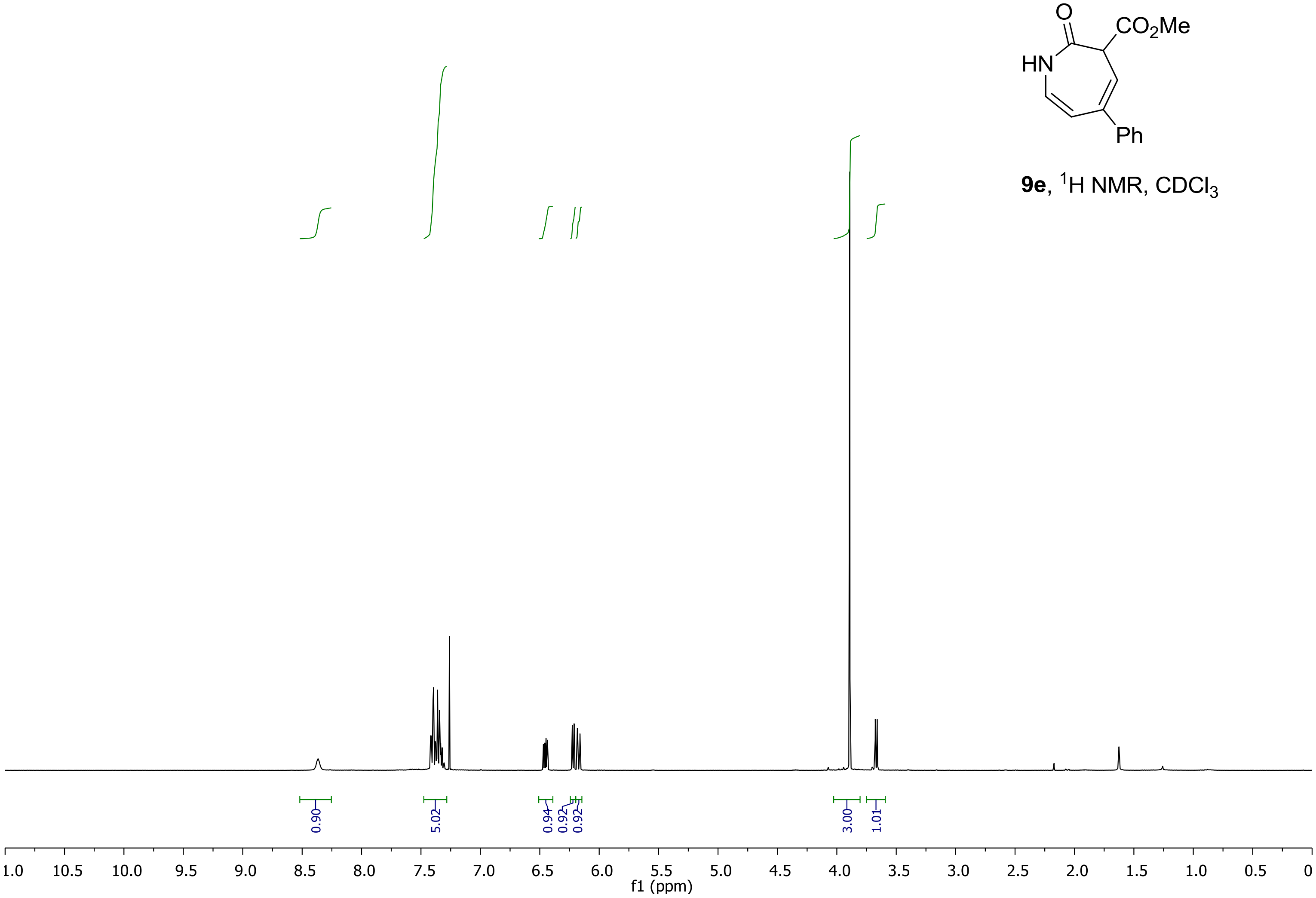

9e, ^{1}H NMR, $CDCl_3$

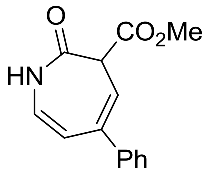

9e, ^{13}C NMR, $CDCl_3$

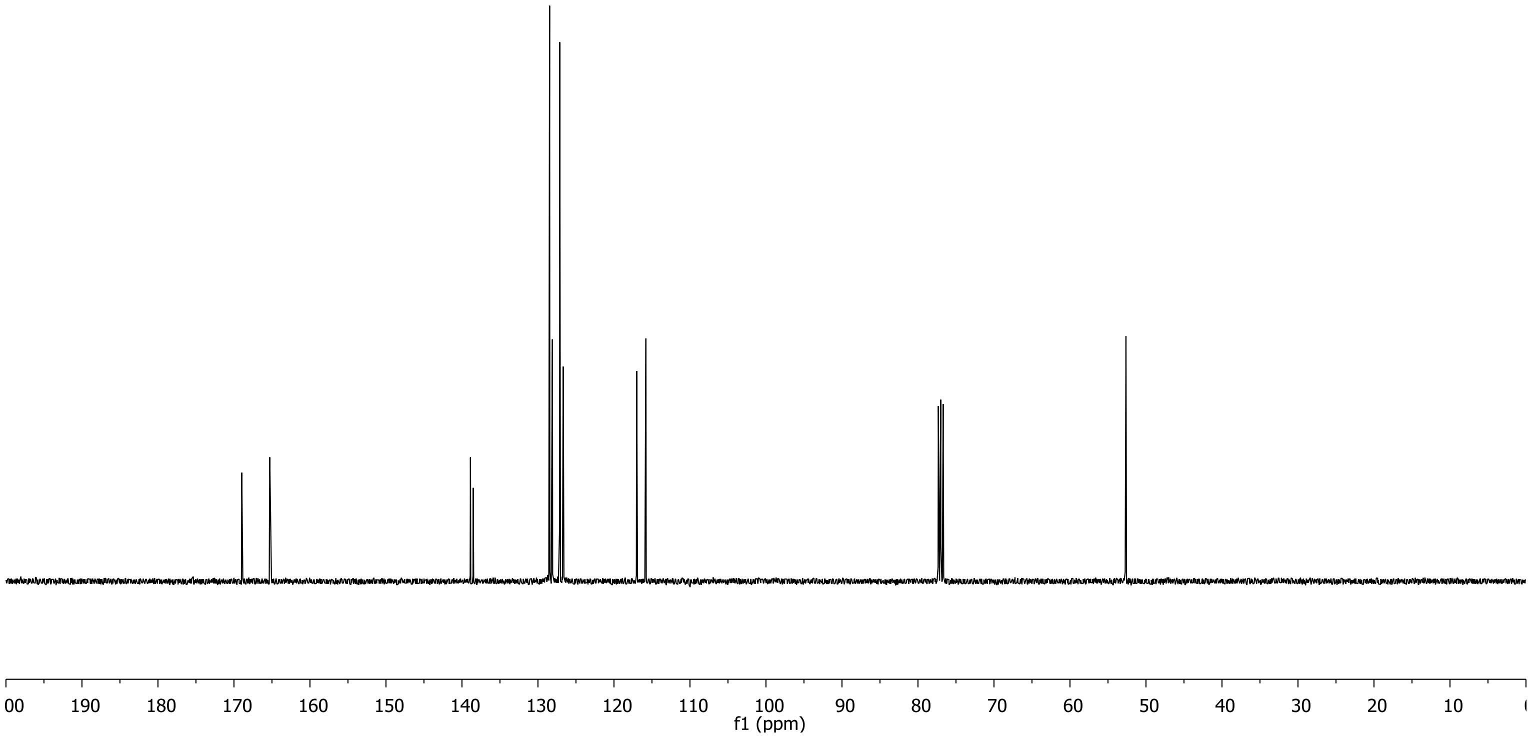

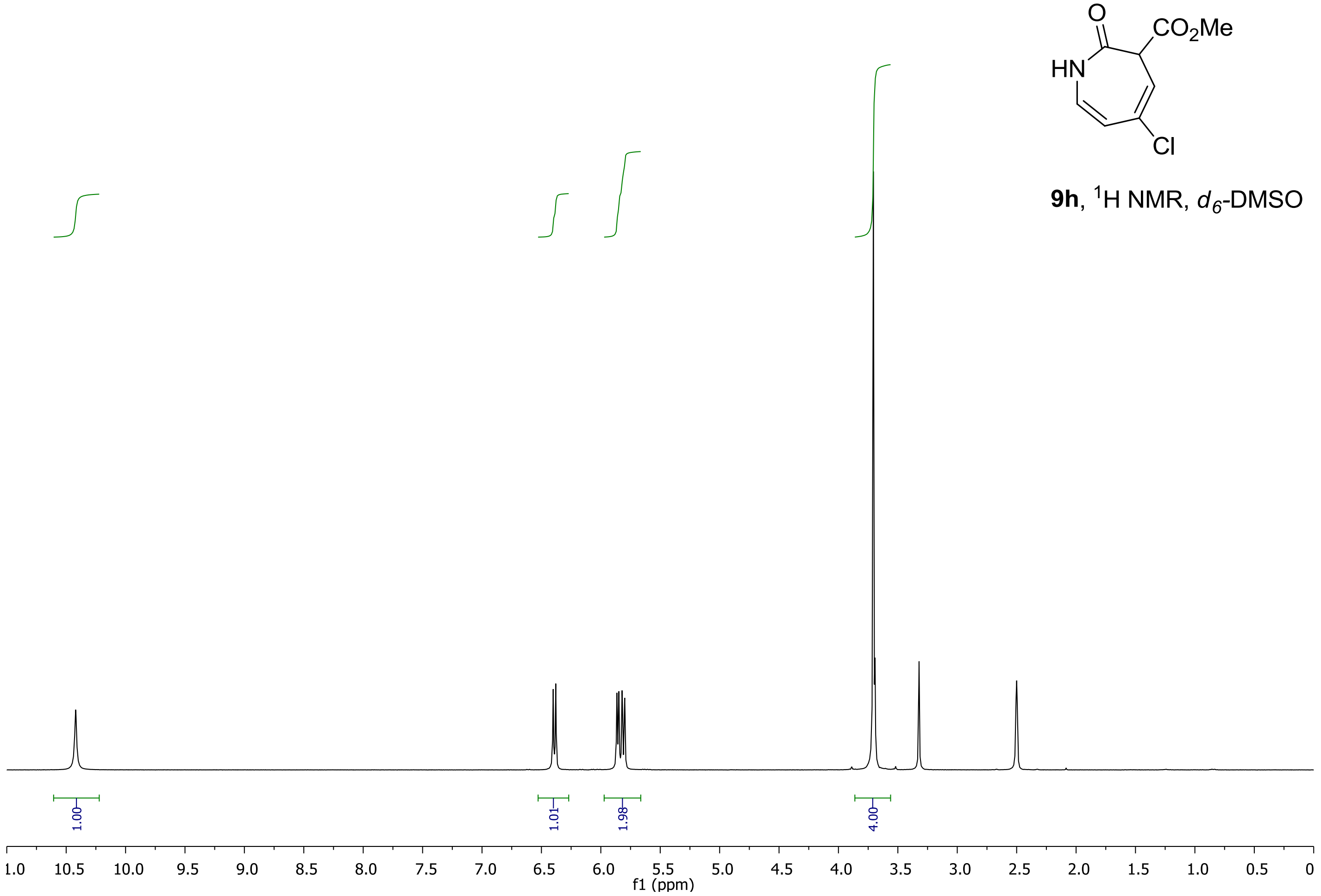

9h, ^{1}H NMR, d_6-DMSO

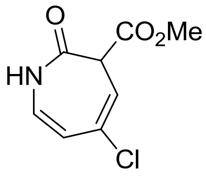

9h, ^{13}C NMR, *d_6*-DMSO

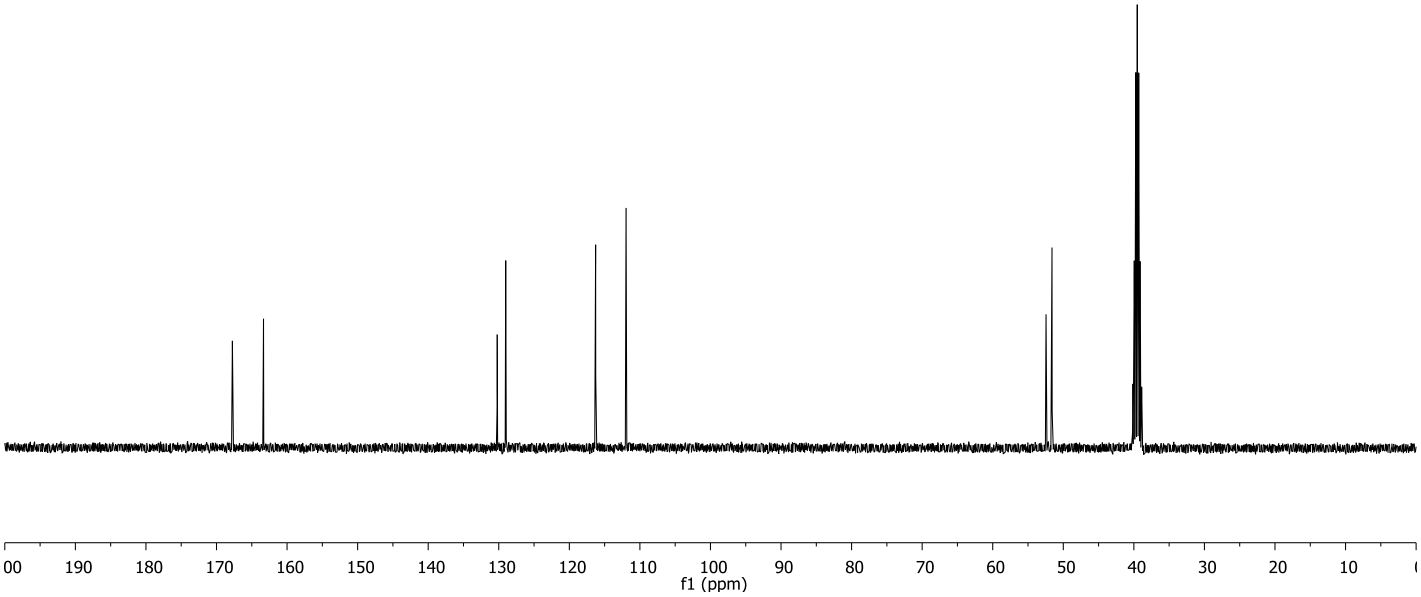

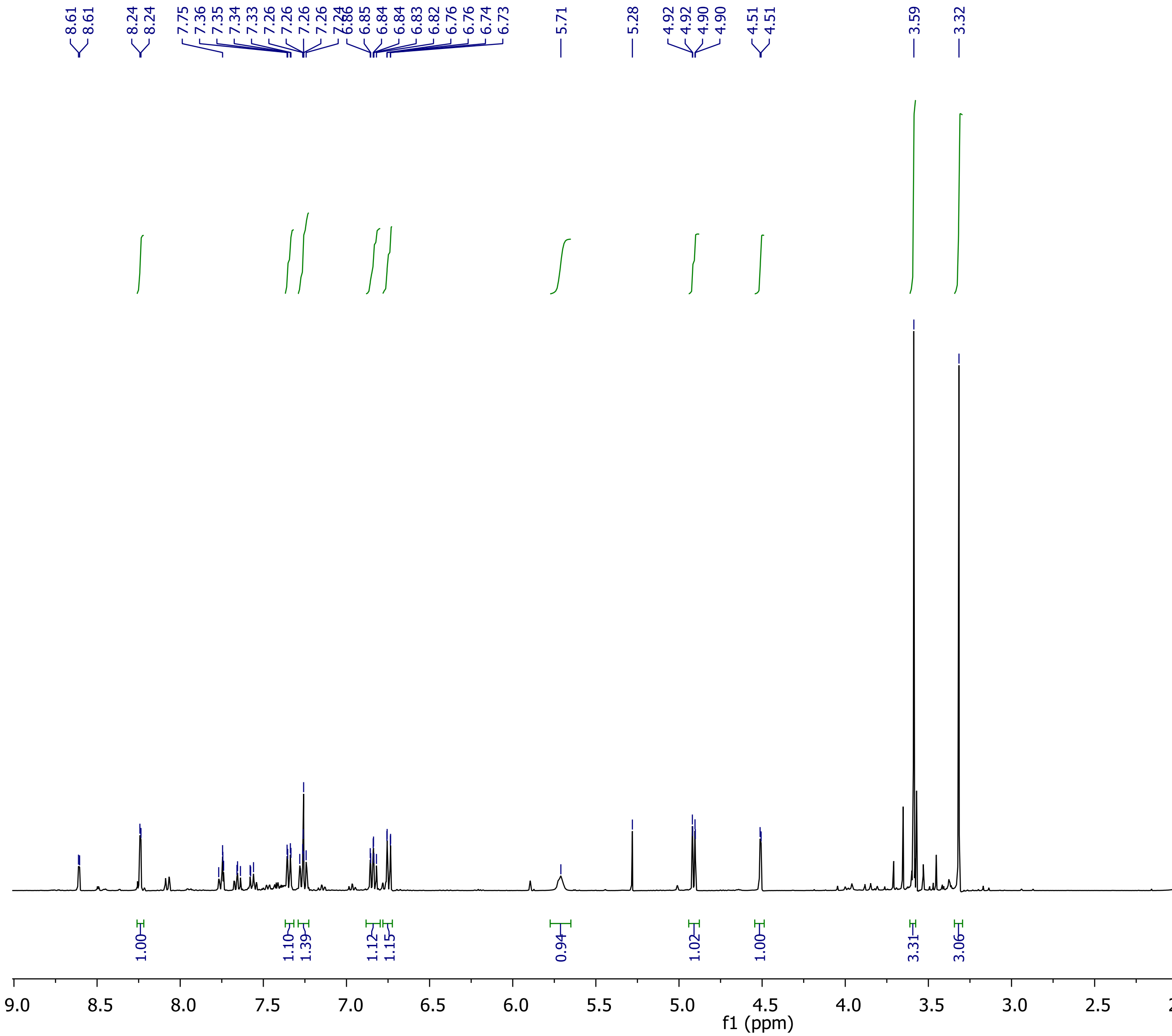

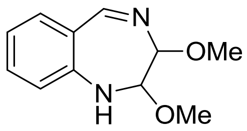

9i, ^{1}H-NMR, $CDCl_3$

including residual starting material **8i**

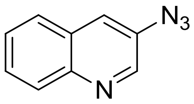

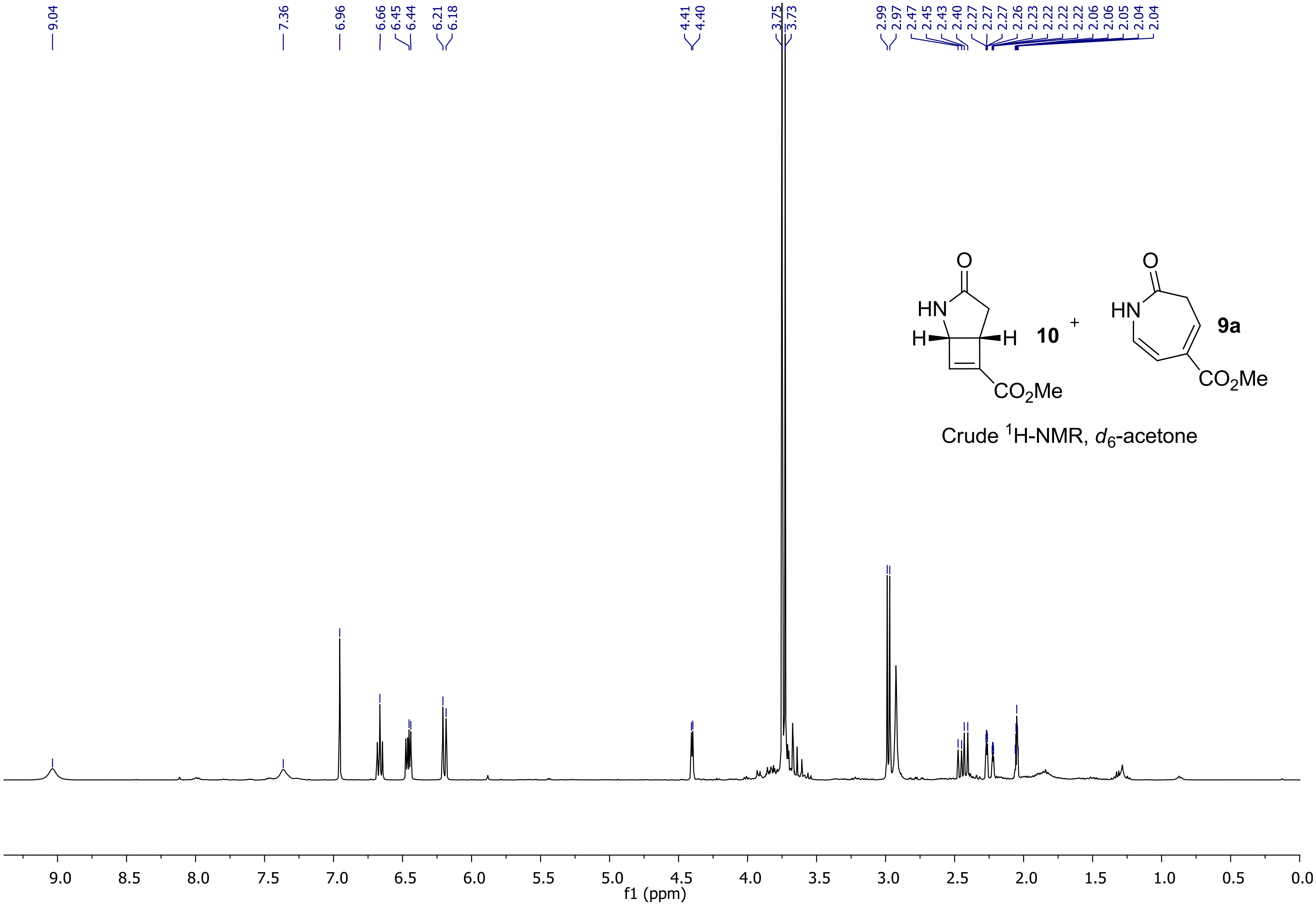
9.04
7.36
6.96
6.66
6.45
6.44
6.21
6.18
4.41
4.40
3.75
3.73
2.99
2.97
2.47
2.45
2.43
2.40
2.27
2.27
2.27
2.26
2.23
2.22
2.22
2.22
2.22
2.06
2.06
2.05
2.04
2.04
O
HN
H
H
10
CO2Me
+
O
HN
9a
CO2Me
Crude 1H-NMR, d6-acetone
9.0
8.5
8.0
7.5
7.0
6.5
6.0
5.5
5.0
4.5
4.0
3.5
3.0
2.5
2.0
1.5
1.0
0.5
0.0
f1 (ppm)

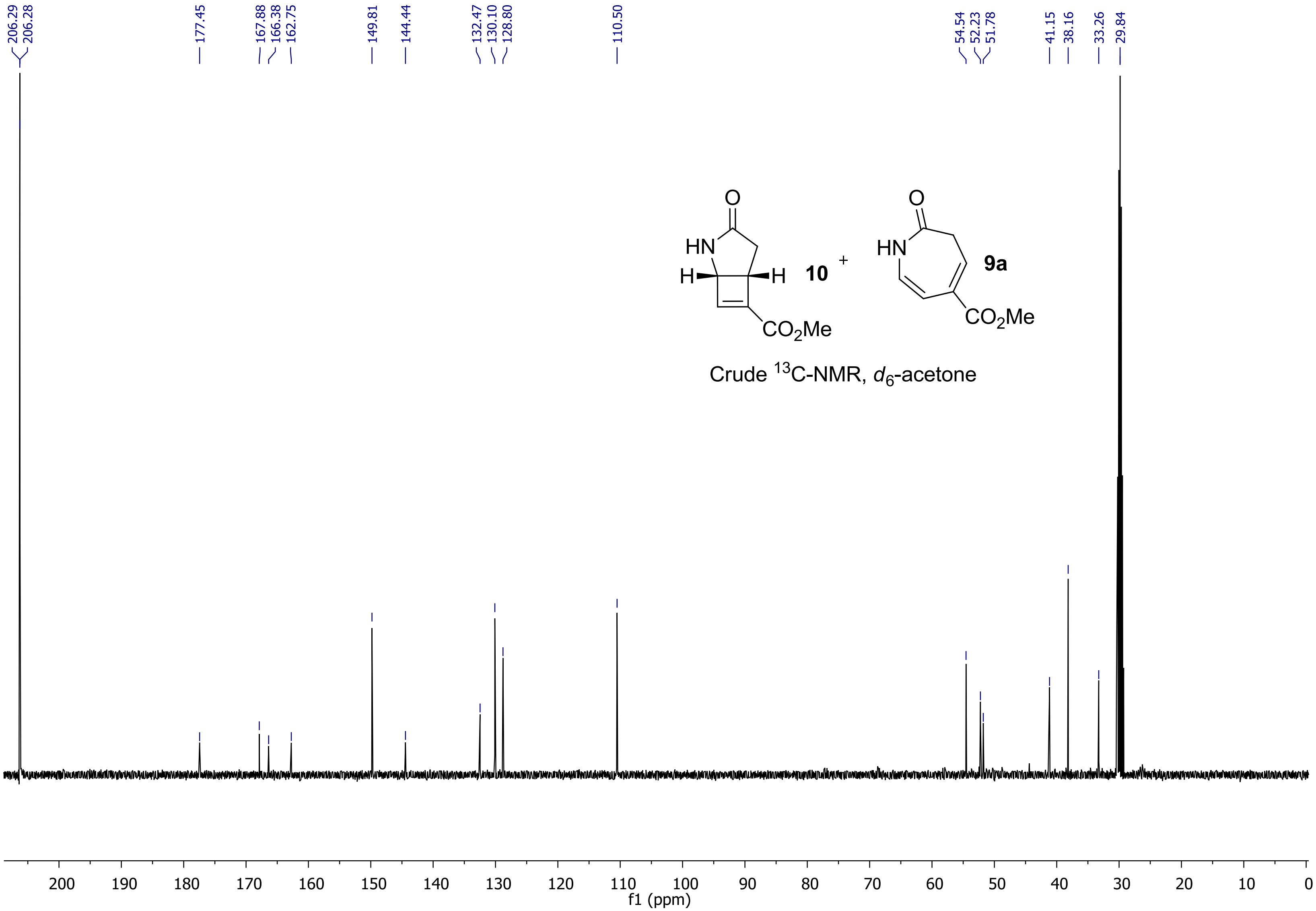
206.29
206.28
177.45
167.88
166.38
162.75
149.81
144.44
132.47
130.10
128.80
110.50
54.54
52.23
51.78
41.15
38.16
33.26
29.84
HN
O
H
H
10
CO2Me
+
HN
O
9a
CO2Me
Crude 13C-NMR, d6-acetone
200
190
180
170
160
150
140
130
120
110
100
90
80
70
60
50
40
30
20
10
0
f1 (ppm)

References

[1] http://www.vapourtec.co.uk/ (accessed July 15, 2011).

[2] http://www.idex-hs.com/ (accessed July 15, 2011).

[3] http://www.aceglass.com/ (accessed July 15, 2011).

[4] Funayama, H.; Sugawara, T. *Bull. Chem. Soc. Jpn.* **1987**, *60*, 2245.

[5] M. Carme Coll Ferrer; Yang, S.; Eckmann, D. M.; Composto, R. J. *Langmuir* **2010**, *26*, 14126.

[6] Morawietz, J.; Sander, W.; Traeubel, M. *J. Org. Chem.* **1995**, *60*, 6368.

[7] Purvis, R.; Smalley, R. K.; Suschitzky, H.; Alkhader, M. A. *J. Chem. Soc.*, *Perkin Trans. 1* **1984**, 249.

[8] Shaw, A. Y.; Chen, Y.-R.; Tsai, C.-H. *Synthetic Commun.* **2009**, *39*, 2647.

[9] Lamara, K.; Smalley, R. K. *Tetrahedron* **1991**, *47*, 2277.

[10] Andersen, H. S.; Olsen, O. H.; Iversen, L. F.; Soerensen, A. L. P.; Mortensen, S. B.; Christensen, M. S.; Branner, S.; Hansen, T. K.; Lau, J. F.; Jeppsen, L.; Moran, E. J.; Su, J.; Bakir, F.; Judge, L.; Shahbaz, M.; Collins, T.; Vo, T.; Newman, M. J.; Ripka, W. C.; Moeller, N. P. H. *J. Med. Chem.* **2010**, *45*, 4443.

[11] Grimes, K. D.; Gupte, A.; Aldrich, C. C. *Synthesis* **2010**, *9*, 1441.

[12] Li, J.; Hu, M.; Yao, S. Q. *Org. Lett.* **2009**, *11*, 3008.

[13] Sashida, H.; Fujik, A.; Tsuchiya, T. *Chem. Pharm. Bull.* **1987**, *35*, 4110.
